# The role of cell‐penetrating peptides in potential anti‐cancer therapy

**DOI:** 10.1002/ctm2.822

**Published:** 2022-05-20

**Authors:** Meiling Zhou, Xi Zou, Kexin Cheng, Suye Zhong, Yangzhou Su, Tao Wu, Yongguang Tao, Li Cong, Bin Yan, Yiqun Jiang

**Affiliations:** ^1^ The Key Laboratory of Model Animal and Stem Cell Biology in Hunan Province Hunan Normal University Changsha Hunan China; ^2^ School of Medicine Hunan Normal University Changsha Hunan China; ^3^ Key Laboratory of Carcinogenesis and Cancer Invasion, Ministry of Education, Department of Pathology, Xiangya Hospital, School of Basic Medicine Central South University Changsha Hunan China; ^4^ Department of Pathology, The Second Clinical Medical College of Jinan University, The First Affiliated Hospital Southern University of Science and Technology Shenzhen People's Hospital Shenzhen China

**Keywords:** Anti‐cancer therapy, cell‐penetrating peptides, molecular cargoes, optimisation, tumour immunity

## Abstract

Due to the complex physiological structure, microenvironment and multiple physiological barriers, traditional anti‐cancer drugs are severely restricted from reaching the tumour site. Cell‐penetrating peptides (CPPs) are typically made up of 5–30 amino acids, and can be utilised as molecular transporters to facilitate the passage of therapeutic drugs across physiological barriers. Up to now, CPPs have widely been used in many anti‐cancer treatment strategies, serving as an excellent potential choice for oncology treatment. However, their drawbacks, such as the lack of cell specificity, short duration of action, poor stability in vivo, compatibility problems (i.e. immunogenicity), poor therapeutic efficacy and formation of unwanted metabolites, have limited their further application in cancer treatment. The cellular uptake mechanisms of CPPs involve mainly endocytosis and direct penetration, but still remain highly controversial in academia. The CPPs‐based drug delivery strategy could be improved by clever design or chemical modifications to develop the next‐generation CPPs with enhanced cell penetration capability, stability and selectivity. In addition, some recent advances in targeted cell penetration that involve CPPs provide some new ideas to optimise CPPs.

## Graphical abstract

1. CPPs are amino acid sequences that can induce uptake processes by the cells.
2. CPPs can facilitate the intracellular delivery of a variety of anti‐cancer therapeutic moleculars.
3. The mechanisms of the CPPs uptake can be generally categorised into energy‐dependent and direct penetration.
4. The optimisation of CPPs' defects will promote their clinical applications as drug delivery vehicles.

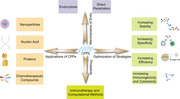

## 1 INTRODUCTION

As a major cause of morbidity and mortality, cancer is threatening human life and health in the world. A study by the GLOBOCAN 2020 estimates indicated that there were 19.3 million new cancer cases and almost 10 million deaths from cancer in 2020. There are quite a few traditional tumour therapies, such as chemotherapy, radiotherapy, targeted therapy, immunotherapy and endocrine therapy. In recent years, to address the problem of effective delivery of exogenous substances from extracellular to intracellular, short peptides carrying drugs and penetrating the cell membrane have attracted extensive attention. Cell‐penetrating peptides (CPPs) can take exogenous substances into cells, and most of them contain less than 30 amino acid residues. CPPs, also known as targeting polypeptides, the protein transduction domain (PTD), and trojan horse peptides,^1^ are derived from natural or non‐natural proteins or chimeric sequences. They can cross the cell membrane, delivering various compounds, such as small molecules, nuclear acids, proteins, viruses, imaging agents and drugs,^2^, ^3^ carrying them into cells in the process. CPPs are usually designed to enhance or inhibit certain specific pathways of intracellular signal transduction. More molecular role refers to improving the anti‐cancer activity, inhibiting ubiquitination, inducing apoptosis and autophagy, mediates host immune responses and so on. As of 28 August 2021, 1855 CPPs have been recorded in the CPPsite 2.0 database. The first protein capable of crossing the cell membrane was discovered by Frankel and Pabo in 1988. When developing a method for detecting the HIV‐1 TAT protein activity, they found that cells cultured in tissue could absorb the purified TAT protein and then reversely activate the viral promoter.^4^ In 1999, Schwarze et al. fused the 120KD‐galactosidase protein with the PTD of the human immunodeficiency virus TAT protein and found that the bioactive fusion protein could be delivered to all tissues of mice, including the brain.^5^ Later, Pooga et al. found that a 21‐peptide PNA complementary to human galanin receptor type 1 mRNA could effectively enter Bowes cells and eventually suppress the expression of galanin receptor when coupled with the CPP transportan or pAntennapedia.^6^ Since then, a large number of CPPs that are able to carry compounds penetrating the cell membrane have been developed by researchers.^7^, ^8^, ^9^ At the cellular level, CPPs have intrinsic anti‐tumour effects (Table 1). Moreover, they can serve as delivery vehicles for different types of cargoes into cells and interact with cell membranes in various ways, such as delivery of chemotherapy drugs or biochemical reagents to the body through their own membrane penetration.^10^, ^11^, ^12^ Surprisingly, some CPPs were found to penetrate the blood–brain barrier (BBB), and the exact mechanisms can be unveiled in their cellular uptake mechanisms.^13^ In addition, featured with low toxicity and efficient membrane permeability, the potential of CPPs can be tapped for a wide range of applications in critical areas such as tumour treatment.^14^, ^15^, ^16^ The use of CPPs, cationic proteins and nano‐carriers were proved an effective path to penetrate BBB,^17^ CPPs combined with transferrin is a classical route targeting for delivery of therapeutic molecules to BBB.^18^ Combining R‐targeting protein with CPP has shown significant progress to overcome BBB as compared to only single ligand receptor‐targeting agents.^19^ Receptor‐mediated transcytosis (RMT) can overcome an impassable penetration to BBB. The ligands such as CPPs, receptor‐targeting peptides and monoclonal antibodies (mAbs) and drugs via nano‐particles (NPs) conjugated with RMT system can induce effective endocytosis to BBB.^13^

**TABLE 1:** Examples of intrinsic anti‐cancer peptide

Name	experiment	Activity	Cancers	Refs.
TP10	In vitro	TP10 improves the anti‐cancer activity of cisplatin, and TP10 also has an anti‐cancer effect on HeLa and OS143B cell lines	Cervical cancer Osteosarcoma	^264^
P28	In vitro	It can penetrate the nuclei of tumour cells and bind to tumour suppressor protein P53 to inhibit ubiquitination.	Breast cancer Colon cancer fibrosarcoma	^265^, ^266^
FK‐16	In vitro	FK‐16 peptide induces apoptosis and autophagy	Colon cancer	^267^
KT2	In vivo	By inducing apoptosis(HCT116)	Colon cancer	^268^
Disruptin	In vitro and in vivo	Inhibition of Hsp90 chaperone and dissociation of active asymmetric EGFR dimer to destabilise activated EGFR		^269^
RALA	In vitro	RALA complex enhanced the tumour growth, delaying activity of alendronate in the PC3 xenotransplantation model of prostate cancer. Retaining pH sensitivity	Prostatic cancer Breast cancer ZR‐75‐1 cell line	^111^, ^270^
TAT	In vitro and in vivo	TAT‐modified pH‐sensitive liposomes significantly reduced cell viability by separating PEG fraction when exposed to the acidic tumour microenvironment, enhancing cellular uptake, delaying tumour growth and prolonging the survival of 4T1 tumour‐bearing BALB/c mice.	Breast cancer	^271^
HNP1	In vitro and in vivo	HNP1 mediates host immune responses to tumours in situ through the recruitment and subsequent activation of immature dendritic cells	Colon cancer Breast cancer	^272^

**FIGURE 1:**
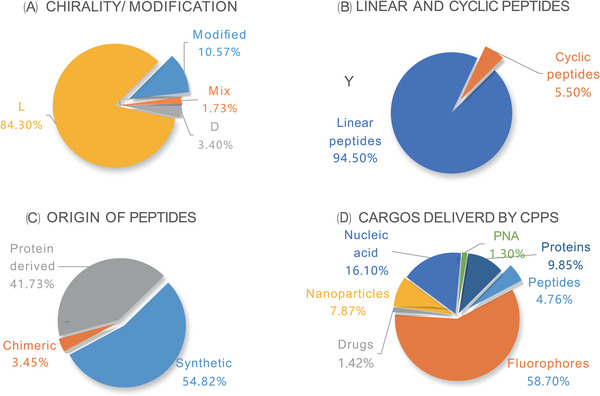
Schematic diagram illustrating the types of CPPs. CPPs are classified in different ways: (A) CPPs can be classified as D type, L type, mix type and modified type based on chirality or modification. (B) According to the conformation, linear peptides are in the majority. (C) CPPs may be derived from different sources, with synthetic CPPs accounting for the largest part. (D) CPPs can be utilised as DDSs and be divided into different subgroups based on their cargoes. Specific classification results and their proportions have been marked in the above figure. The most popular classification is according to physical–chemical properties, in which CPPs can be classified into three subgroups: cationic CPPs, amphipathic CPPs and hydrophobic CPPs

**FIGURE 2:**
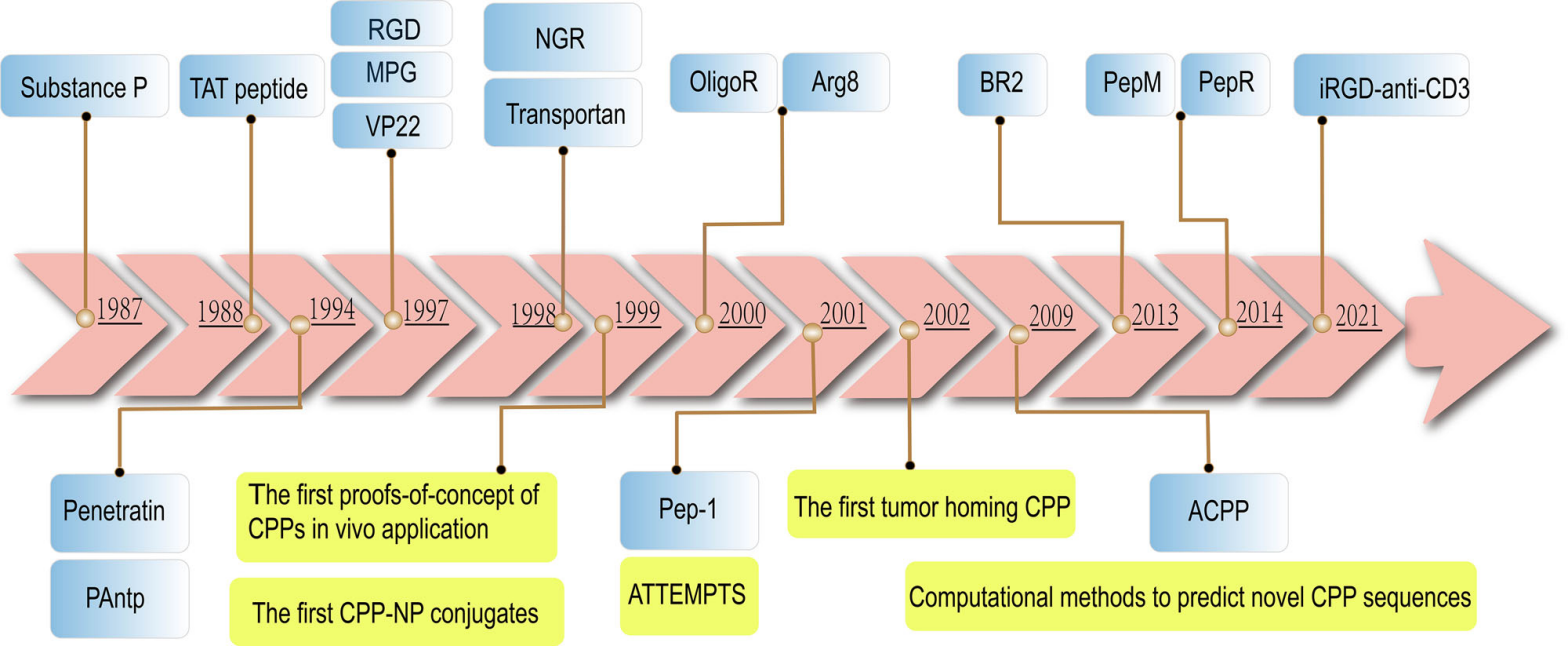
Chronological arrow in CPP development. Those in the blue box represent important CPPs with epoch‐making significance, and those in yellow are major events. ATTEMPT, ACPP and tumour homing CPPs can be seen in Part 4

CPPs of different origins or compositions vary greatly in physical, chemical and biological properties, manifested as differences in length, charge, solubility and hydrophobicity. At present, CPPs are usually classified according to their source, conformation and physical and chemical properties. The most common classification is cationic, lipophilic and hydrophobic CPPs, in terms of their physical and chemical differences. They can be divided into D‐type, L‐type, mixed type and modified type based on chirality/modification. Moreover, CPPs can be classified according to the cargoes they transport. The classification basis and the proportion of each category are shown in Figure 1 (https://webs.iiitd.edu.in/raghava/cppsite/stats1.php). This paper focuses on the specific research or application of CPPs combined with different drugs to treat related tumours in recent years and some significant defects confronted by current CPPs against the further progress of anti‐tumour treatment based on CPPs. More importantly, the timeline of some well‐established CPPs is shown in Figure 2. There has been new progress in promoting CPPs’ tumour therapy by delivering nucleic acids (NAs) and proteins, interacting with ligands or receptors and combining NPs. Combine with CPP‐Dot 1l or incubation with DMSO is another method to synergistically promote the delivery of plasmid DNA mediated by 36+GFP.^20^ CPP‐CaaX can affect K‐Ras downstream signalling to promote death of tumour cells, especially K‐Ras‐4B.^21^ CPPs containing the progesterone receptor polyproline domain can inhibit cell proliferation in lung cancer.^22^ CPP‐modified graphene oxide NPs loaded with Rictor siRNA significantly inhibited TNBC progress by inhibiting PI3K/Akt/mTOR signal transduction.^23^

## 2 THE UPTAKE MECHANISM OF CPPs

**FIGURE 3:**
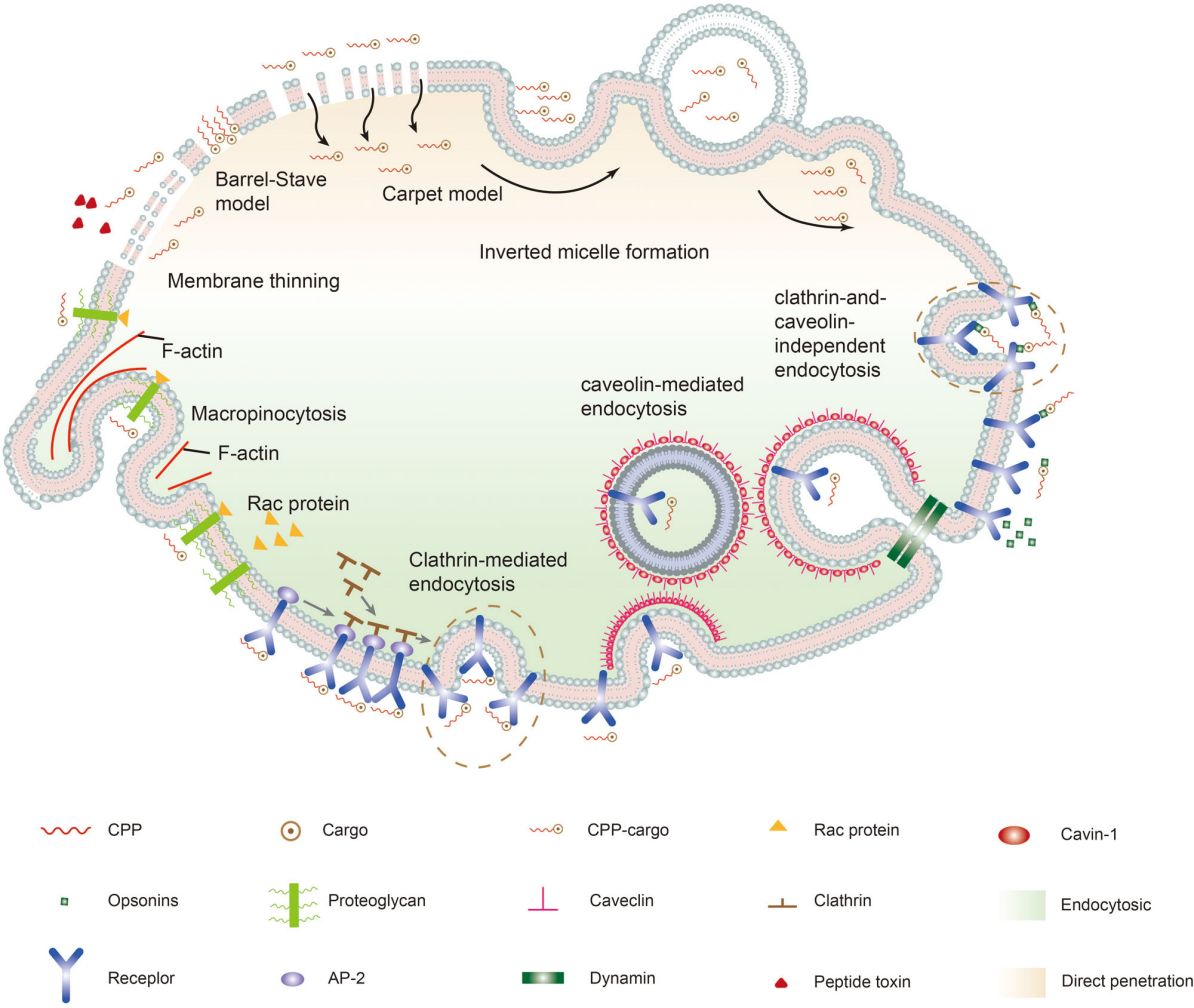
Mechanisms of the intracellular entry of CPPs. Schematic representation of mechanisms for CPPs internalisation. The involved pathways can be divided into two groups: endocytosis (blue) and energy‐independent mechanisms (pink). Endocytosis pathways consist of macropinocytosis, caveolin‐mediated endocytosis, clathrin‐mediated endocytosis and clathrin and caveolin‐independent endocytosis. Energy‐independent mechanisms have been proposed to occur through: the ‘barrel‐stave’ model, the ‘carpet‐like’ model, the inverted micelle model, the membrane thinning mode and another hypothesis of ‘membrane thinning’. The small molecules involved in the uptake progress have been marked in the domain of the relevant pathway

The cellular uptake mechanism of CPPs remains highly controversial in academia. Although the mechanism of cellular uptake is far from being fully understood, it is speculated that there may be several independent mechanisms for cellular uptake of CPPs or many mechanisms working together, according to the different physicochemical properties of CPPs. In addition, many researchers also claimed that the cellular uptake mechanism of CPPs depends on the type, size, concentration and charge of the carriers.^24^ A better and more comprehensive understanding of the cellular uptake mechanism will contribute to the CPPs‐based research and application. Currently, the acknowledged cellular uptake mechanisms of CPPs mainly contain endocytosis‐mediated and energy‐independent pathways, as shown in Figure 3. More information about the uptake mechanism of CPPs can be found in the review.^24^

The penetration ability of CPPs was related to their net charge and peptide length to some extent. Peptides with positive net charge have better cell membrane penetration ability than those with negative net charge or no net charge.^25^ The more efficient uptake of CPP with cation may be explained by the presence of anionic proteoglycans and phospholipids on the cell membrane, which leads to the electrostatic interaction between the cationic CPPs and the membrane for internalisation. It is proved that CPPs with a sequence length of more than 12 amino acid residues had better penetration ability than shorter peptides. In addition, peptides with a higher helix orientation tend to have greater penetration ability.^26^ Molecular dynamics simulations have demonstrated that the identified benzene dithiol linker can greatly facilitate the α‐helicity of a variety of peptide substrates to enhance the penetration ability of CPPs.^27^ Due to the instability of the helix structure of the short peptide in solution, it can be speculated that peptides with sequence lengths less than 13 amino acids are insufficient to maintain a stable alpha‐helix conformation. The hydrophobic interaction of CPPs also has a positive effect on the penetration ability of CPPs. The aromatic tryptophan can be considered hydrophobic, and the penetration ability of CPPs is reported to be positively correlated with the number of tryptophan residues.^28^ Arginine residues play an important role in cell membrane interactions, uptake mechanisms and hydrophobic α‐helix structures, which was demonstrated by the comparison of the delivery efficiencies of TP10 and Pen‐Arg.^29^ At the same time, the concentration of CPP affected the uptake mechanism of CPP, which means that high concentrations of primary hydrophobic CPP were more likely to penetrate cell membrane directly, whereas endocytosis was the primary absorption mechanism at the low concentrations.^30^ Researchers have found that the permeability enhancer can be used to improve the penetration ability of CPPs. The permeability efficiency of 36+GFP protein can be remarkably improved by fusing with CPP‐Dot1l or treating with the permeability enhancer dimethyl sulfoxide (DMSO) in vitro.^20^

### 2.1 The endocytosis‐mediated pathway

Endocytosis is regarded as an internalisation process of peptides or macromolecules. The endocytosis‐mediated pathway, also known as the energy‐dependent pathway mechanism, serves as the dominant cellular uptake pathway for most CPPs.^31^ As a natural and energy‐dependent process, the endocytosis‐mediated pathway exists in all cells. There are four identified pathways: macropinocytosis, caveolin‐mediated endocytosis, clathrin‐mediated endocytosis and clathrin‐ and caveolin‐independent endocytosis.

Macropinocytosis is a rapid receptor‐independent cellular uptake process that involves the formation of membrane protrusions driven by actin polymerisation.^32^, ^33^ The process of macropinocytosis includes the interaction of CPP/cargoes and membrane proteoglycans, which activates the rac protein in the cytoplasm. Then, the rac protein releases signals to stimulate the F‐actin organisations, followed by the contraction of actin microfilaments, the deformation, protrusion and endocytosis of cell membrane, and then the cellular uptake of CPPs.^34^ Clathrin‐mediated endocytosis, sometimes called as receptor‐mediated endocytosis, is receptor‐guided and clathrin‐mediated endocytosis necessary for the cellular uptake of essential nutrients.^35^ Its mechanism involves the following processes. Firstly, CPPs interact with receptors and induce the clathrin assembly to form a new cytoplasmic membrane. The membrane then undergoes progressive clathrin‐coated invagination and eventually forms clathrin‐coated vesicles with a size of 150 nm. After that, the clathrin‐coated vesicles are converted into endosomes and then released into the cytoplasm.^36^ Similar to clathrin‐mediated endocytosis, caveolin‐mediated endocytosis occurs, whereas the difference lies in the fact that the former is associated with caveolin. Caveolin‐mediated endocytosis is a clathrin‐independent process concerning the formation of intracellular vesicle invagination and is mediated by the membrane protein caveolins and the peripheral protein cavins.^37^ In this mechanism, to induce endocytosis, CPP/cargoes specifically target the receptors on lipid rafts, which are hydrophobic areas rich in cholesterol and sphingomyelin (also known as lipid rafts).^38^ Cavin connects to caveolin, which then will generate and invaginating a pit as cavin and caveolin complexes increases gradually. Then cell takes in a caveolin‐coated vesicle to form an endosome. Caveolin is the main surface marker protein and exists predominantly in the vesicles, Golgi apparatus, and partially soluble lipoprotein complexes on the membrane. It is a membrane integrin that functions as a molecular signalling platform. Studies have shown that the mechanisms of these three endocytosis pathways are minimally affected by the dose and sequence of CPPs.^39^ Different from the above‐mentioned pathways, clathrin‐ and caveolin‐independent endocytosis mainly occurs in animal cells for internalisation of fluids, membrane components and receptor–ligand complexes.^40^ Additionally, opsonins can also recognise and label CPP/cargoes, and the process is as follows: CPP/cargoes firstly attach to the Fc receptor on the cell membrane, activate actin and subsequently are coated by the cell membrane. Then, CPP/cargoes translocation is complete.^41^

### 2.2 Direct penetration

Direct penetration, also referred to as the non‐endocytosis pathway or energy‐independent pathway, is a cellular uptake process independent of energy. This mechanism is based on the interaction of cationic CPPs with the anionic components of the cell membrane, such as heparan sulphate (HS) and the phospholipid bilayer. The interaction later leads to temporary destabilisation of the plasma membrane via or pore formation, which usually happens at low temperatures without the participation of receptors. According to the differences of corresponding uptake mechanisms, they are proposed to occur through the ‘barrel‐stave’ model, ‘carpet‐like’ model, inverted micelle model and membrane thinning model.

The ‘barrel‐stave’ model (also known as the pore formation model) describes an antibacterial peptide that destabilises the bacterial cell membrane, which undergoes depolarisation after being destabilised by the antibacterial peptide.^42^ In this mechanism, some pores form on the surface of the cell membrane. When amphiphilic CPPs or CPP/cargoes penetrate the cell membrane, their hydrophobic regions interact with the phospholipids on the cell membrane, and their hydrophilic sections combine with the hydrophilic heads of phospholipids to produce barrel‐shaped pores, facilitating transmembrane mobility.^43^, ^44^ Thus, CPPs can penetrate the cell membrane and enter the cytoplasm.

The ‘carpet‐like’ model is also described as an antibacterial peptide translocation model, featured by a transient increment in membrane fluidity.^45^ CPPs or CPP/cargoes penetrate the cell membrane via charge interaction in the ‘carpet‐like’ model.^46^ CPPs or CPP/cargoes cover the surface of the cell membrane in a carpet‐like pattern, with their hydrophobic sections interacting with the cell membrane's hydrophobic regions.^33^ When the concentration of CPPs or CPP/cargoes reaches a certain level, their hydrophobic parts would be overturned by the cell membrane's hydrophobic core, increasing cell membrane mobility. The membrane thus becomes locally destabilised, expediting the delivery of CPPs and their cargoes into cells.

To describe the transmembrane protein family of CPPs, the inverted micelle model has also been proposed.^47^, ^48^ Transmembrane protein CPPs are polypeptides rich in lysine and arginine that can penetrate the cell membrane at low temperatures, indicating that no energy is required for the process. According to the hypothesis, cationic residues (arginine and lysine) firstly combine with anionic phospholipids on the cell membrane^49^ to form pocket micelles that encapsulate CPPs.^50^, ^51^ Next, CPPs penetrate the cell membrane in the micelle towards the cytoplasmic side. When these micelles cross the membrane, CPPs and their carriers are released into cells.

The ‘membrane thinning’ effect of alternative carpet models was initially suggested using a peptide toxin, magainin.^52^ In this model, anionic lipids are laterally rearranged, which is caused by external charge interactions, and then the membrane becomes thinner. The interaction with CPPs and the membrane surface reduces the local surface tension and allows the insertion of CPPs into the membrane, thus achieving effective penetration of CPPs. Moreover, after the internalisation of CPPs onto corresponding targets, the membrane will reseal naturally.

To be more specific, uptake pathways of CPPs depend on their properties, concentration, type of cargo, cell lines used and experimental conditions. At such a circumstance, the same experiment may obtain different results regarding the uptake mechanisms, so a better understanding of the role of these factors and standardisation of the experimental methods will undoubtedly lead to the agreement of uptake mechanisms of CPPs.

## 3 CPPs AND ANTI‐CANCER CARGOES DELIVERY

In recent years, ‘precision medicine’ has received increasing attention in tumour treatment. The combination of CPPs, other drugs such as chemotherapy drugs, NA molecules, peptide NAs, proteins, peptide vaccines and NPs, greatly expands the application prospect of CPPs in tumour treatment. The anti‐cancer applications or pre‐clinical studies described in this section are mainly shown in Table 2.

**TABLE 2:** Some CPPs‐based experiments

	CPPs	Other conjugates	Cargoes	Tumour/cell lines/animal model	experiment	Refs.
Chemothera‐peutics delivery	PEGA‐pVEC peptide	–	EGCG	Breast tumour‐bearing mice	In vivo	^11^
	KRP	–	Dox	Osteosarcoma MG63	In vitro	^273^
	R8	–	Oxaliplatin	Colon cancer	In vitro and in vivo	^169^
	R7	Cyclodextrin and PLGA	DTX	Breast cancer	In vitro	^162^
	SCPP‐PS	–	MTX	Mice bearing A549 lung tumour xenografts	In vitro and in vivo	^172^
	LMWP‐TAT	–	PTX	A549 and A549T cells	In vitro	^157^
	R7	–	PTX	HeLa cells	In vitro	^158^
siRNA delivery	R9	–	Anti‐Polo‐ like kinase 1	Breast tumour	In vitro and in vivo	^274^
	TAT‐A1	–	Anti‐GAPDH	Co‐cultured tumour cells	In vitro	^275^
Peptide delivery	LMWP	–	gelsolin	The 293T, HeLa human cervical carcinoma, CT‐26 colon adenocarcinoma cell line, human MCF‐7 breast carcinoma cell lines, MG 63 osteoblast cell line and NIH3T3 fibroblast cell lines	In vitro and in vivo	^276^
	Antp‐BH3	–	Bcl‐2	HeLa cells	In vitro	^277^
	TAT	–	Gelonin toxin,anti‐CEA mAb	LS174T and HCT116 human adenocarcinoma cells, MDCK (Madin‐Darby canine kidney) and 293 HEK (human embryonic kidney) cells	In vitro and in vivo	^112^
	Penetratin	–	KLA peptide, (KLAKLAK)2	The non‐small cell lungcancercellline (A549) and theneuroblastoma 153cell line (SK‐N‐SH)	In vitro	^278^
	Transportan 10	–	LXXLL motif of the human SRC‐1 nuclear receptor box 1	Breast cancer	In vitro	^279^
	Bac	–	p21Waf/clip‐derived peptide	Pancreatic tumour cells	In vitro and in vivo	^280^
	PNC‐21, PNC‐27, PNC‐28	–	Pen	A549 human lung non‐small‐cell carcinoma cells, HeLa human cervical carcinoma cells, p53‐null SW 1417 cells, human metastatic colon adenocarcinoma cells	In vitro	^281^
NPs	MCaUF1‐9(Ala)	–	GNPs	HeLa, MDA‐MB‐231 and A431	In vitro	^282^
	PEG	Herceptin (HER)	GNPs	Breast cancer	In vitro and in vivo	^66^
	TAT	Protoporphyrin IX	GNPs	BT‐549 breast cancer cells	In vitro	^283^
	TATp, Other NLS peptides	–	GNPs	NIH3T3, HepG2, HeLa, hTERT‐BJ1	In vitro	^284^
						^285^
	TATp	–	Quantum dot loaded micelles	MS1, lineage‐negative bone marrow cells	In vitro	^285^
	Angiopep‐2,TAT	DTX	Nano‐micelles	Glioma cell	In vitro and in vivo	^73^
	TATp	–	pH‐sensitive PEG polylactic acid micelles	MCF‐7	In vitro	^286^
	AP peptide	DOX	pH‐sensitive PEG polylactic acid micelles	breast cancer	In vitro and in vivo	^287^
	R8	siRNA	liposomes	NCI‐H446, A549, SK‐MES‐1	In vitro	^288^
	R8	Doxorubicin	liposomes	Non‐small cell lung cancer cell line, A549	In vitro	^289^
	R8‐RGD	PTX	liposomes	Glioma	In vitro and in vivo	^220^
	TATp, Penetratin and Octa‐ arginine	–	liposomes	Calu‐3	In vitro	^290^
	TAT, Penetratin	Doxorubicin	liposomes	SK‐BR‐3, MCF‐7, HTB 9, ADR, A431,C26	In vitro and in vivo	^291^
	TH peptide	PTX	liposomes	C26 tumour model	In vitro and in vivo	^292^
	TAT	–	MWCNTs	Human breast cancer cell line MD‐MBA‐231 cells	In vitro and in vivo	^89^
	R5W3R4, R9	–	AgNPs	MCF‐7 cell lines	In vitro	^93^

### 3.1 Delivery or conjugation with NPs

For the past few years, with the rapid advances in nanoscience and technology and remarkable achievements in the medical field, a nano‐based diagnostic and therapeutic system has broadened a new strategy for cancer. Nano‐medicine products offer opportunities to realise complex targeting and develop multifunctional therapeutic strategies. Overall, the applications of NPs in clinical practice are confronted with barriers due to their shortcomings. For example, in vivo, the intracellular delivery efficiency of NPs is far from desirable so that they cannot effectively cross the BBB.^53^, ^54^ Despite the unknown uptake mechanisms of CPPs/CPP‐cargoes, many CPPs have been used to enhance intracellular delivery of various NPs efficiently, as evidenced by several excellent reviews. In 1999, Josephson et al.^55^ were the first to disclose the conjugation of CPPs to NPs. The coupling of NPs and CPPs can reduce the side effects of anti‐cancer drugs, enhance the specific cellular uptake and extend the cycle time, providing controlled release and package for multiple drugs in combination therapy. In addition, CPPs can also bind to transport drugs to form nano‐micro molecules, in which efficient transport can also be achieved. The newly formed NPs have considerable advantages, such as good biocompatibility, low cytotoxicity, high cell membrane permeability, large surface area, easy modifiability and abundant functional groups on the surface.^56^, ^57^, ^58^, ^59^ CPPs, as a functional material, may address not only the issue of geographical localisation, but also the issue of intracellular internalisation. CPPs’ role of modifying several polymers to enhance their cell‐penetrating capability is also indicated in the CPPs and NPs conjugation strategies, where the complex interplay (which can be mainly divided into two types—covalently and non‐covalently) between CPPs and NPs eventually allows for their implementation in various fields such as cancer targeting, imaging and therapy. Moreover, the coupling of CPPs to the surface of NPs has also been shown to improve the tumour‐targeting ability of NPs.^60^ Compared with CPPs‐mediated NPs, NPs modified by targeting molecules (such as hyaluronic acid and folic acid) have their own characteristics. Depending on the specificity of targeting molecules, NPs modified by them may exhibit higher selectivity. As targeting molecules are mainly naturally occurring compounds, such polysaccharide‐coated NPs can increase their biocompatibility, but the attachment of CPPs to NP surfaces is much more convenient. In addition, X‐ray + TAT‐GNSs treatment was confirmed a distinct tumour inhibitory ability, which illustrated the highest radiation sensitising ability of TAT‐GNSs.^61^ In general, some of the most popular CPPs‐NPs combinations were described in this section.

#### 3.1.1 Gold nano‐particles

In 1857, Faraday published the first scientific article about gold NPs (AuNPs). For centuries, AuNPs with sizes ranging from 1 to 120 nm have been recommended to treat various diseases. Due to some significant properties of AuNPs, such as adjustable local surface Plasmon resonance, good surface modifiability and biocompatibility, easy synthesis and stable properties, AuNPs have long been thought to have significant potential in cancer diagnostics and medication delivery applications, according to researchers. In addition, the non‐toxicity and non‐immunogenicity of AuNPs and the enhanced permeability and retention (EPR) effect can make therapeutic drugs permeate and accumulate more easily at the tumour site, providing an additional benefit for therapeutic use.^62^ Additionally, their nanometre size as well as magnetic and luminescent properties are adequately bodied in the application of AuNPs in cancer diagnosis and treatment.^63^, ^64^

As for the combination of CPPs and NPs, the former can be used not only to improve the intracellular delivery of GNPs but also to reduce their cytotoxicity.^65^ Functionalised GNPs have been showed no cytotoxicity against host cells. A complex of covalently bound GNPs to PEG and the mAb Herceptin were found to recognise breast cancer cells expressing specific tumour‐related antigens and had better stability and activity in vivo and in the blood of a nude tumour xenograft‐bearing mouse model.^66^ Lupusoru et al.^67^ synthesised TAT‐DOX‐PEG‐irradiated AuNPs conjugates to investigate the cytotoxic effect on tumour cells of irradiated AuNPs in green light, followed by functionalisation with HS‐PEG‐NH2. A cell viability test proved that irradiated and non‐irradiated NPs coated with PEG were non‐toxic to normal cells. The results indicated that the PEGylated NPs modified with DOX and TAT peptides were more effective than pristine DOX. To enhance drug delivery to inhibit breast cancer cells, Ramin A. Morshed et al.^68^ designed a cell‐penetrating GNP platform of TAT peptide‐modified AuNPs carrying DOX. When compared to free drugs, cytotoxicity to two breast cancer cell lines was significantly improved as evidenced by noticeable reduction in IC50. Furthermore, in vitro investigations revealed substantial particle accumulation in tumour foci and enhanced survival in an intracranial MDA‐MB‐231‐Br xenograft mouse model.

#### 3.1.2 Nano‐micelles

According to the polarity and non‐polarity of the solution, nano‐micelles can be classified into common nano‐micelles and reverse nano‐micelles.^69^ Due to inherent and easily modified characteristics, nano‐micelles are suitable for drug delivery, especially when used with CPPs. Additionally, CPPs can promote the penetration of nano‐micelles into cells to achieve better therapeutic effects.^70^ The use of CPP‐conjugated polymer micelles is becoming an increasingly hot topic under investigation in the field of tumour‐targeted therapy. For example, researchers have successfully applied micelle systems containing TAT peptides to specifically deliver anti‐cancer drugs to solid tumours and found promising experimental results.^71^, ^72^

Ya qin Zhu et al.^73^ proved that tandem nano‐micelles could achieve efficient and specific anti‐glioma chemotherapy effects if they are co‐functionalised with the brain tumour‐targeted CPPs Angiopep‐2 and TAT. The complex not only showed high selectivity to glioma cells and a long blood circulation time, but also enhanced the penetration effect of BBB. This experiment powerfully demonstrates that the tandem nano‐micelle loaded with DTX could achieve a better anti‐tumour effect in situ U87MG human gliomas without significant adverse effects than the single peptide‐functionalised counterpart of Angiopep‐2.

In addition, we have noticed that the PH‐targeting system is becoming increasingly popular in anti‐cancer therapy. That system can specifically release the carried substances based on the specific pH in the tumour microenvironment. In other words, there is no release at pH 7.4, but when it enters the tumour microenvironment (pH 5.5–6.5), a complete release can be observed.^74^ This remarkable pH‐specific release mechanism can be exploited to develop targeted delivery systems for anti‐cancer drugs. Jingwen Shi et al.^75^ synthesised RGD‐decorated PEG‐PTX self‐assembly micelles to achieve targeted therapy against carcinoma of stomach. The growth of carcinoma of stomach cells was significantly inhibited by the conjugate. The conjugate's tumour targeting and inhibitory efficacy was validated in a carcinoma of stomach xenograft mouse model.

#### 3.1.3 Liposomes

Liposomes with phospholipid vesicle structures were first discovered in the 1960s. They are hollow bilayer structures formed by one or more concentric lipid bilayers. Liposomes can be degraded rapidly during systemic circulation due to their size, surface charge, hydrophobicity and fluidity of the lipid membrane.^76^, ^77^, ^78^ Researchers modified liposomes with PEG to avoid their recognition by the RES system, which showed increased liposomal stability, prolonged circulation half‐life, improved biodistribution profile and enhanced anti‐cancer potency of the drug payload. In conclusion, their surface can also be modified by attaching CPPs.^79^ Compared with other drug delivery systems (DDSs), liposome‐related ones exhibit many significant characteristics, such as local compatibility, low immunogenicity and self‐assembly capabilities. In addition, they are found to carry macro‐molecular drugs, protect the delivered drugs from external media and simultaneously load hydrophilic drugs and hydrophobic targeting ligands. They are also found to reduce the toxicity of transport drugs, avoid toxic drugs from damaging sensitive tissues of the body and possess a better site‐specific targeting and enhanced drug penetration ability.^80^, ^81^, ^82^, ^83^ The use of CPPs to modify the surface of liposomes has received increasing attention because their combination allows for superior cell permeability.^84^

Biswas et al.^83^ functionalised DOX‐loaded PEGylated liposomes (commercially available DOXIL or LipoDOX®) with CPPs octaarginine (R8) . According to results, this simple surface modification of liposome‐DOX can resolve the poor permeability of PEGylated liposomes and provides a strategy to significantly boost their anti‐cancer activity. Many drug delivery examples of the combination of CPP and liposome are found to help overcome the obstacle of BBB for a better therapeutic effect on neurologic tumours. Yayuan Liu et al.^85^ conjugated the RGD reverse sequence dGR to R8 and synthesised a CendR motif contained tandem peptide R8‐dGR. The dual receptor (integrinαvβ3 and neuropilin‐1 receptors) recognising peptide R8‐dGR showed increased cellular uptake and efficient penetration ability into glioma spheroids in vitro. When PTX was loaded into liposomes, PTX‐R8‐dGR‐Lip could induce the most substantial anti‐proliferation effect and inhibit angiogenesis in vitro. The CPP‐modified liposomal formulation can show significantly higher tumour suppression efficacy with minimum collateral toxicity.

#### 3.1.4 Carbon nanotubes

Carbon nanotubes (CNTs) are considered to be a very potent drug delivery vehicle for small molecules and biologics due to their properties, such as high surface area to volume ratio, superior efficacy, enhanced specificity and mild side effects.^86^ To our knowledge, CNTs are widely applied in tumour treatment.^87^, ^88^ However, the further application of CNTs in cancer treatment is limited by the lack of water solubility and cytotoxicity due to their hydrophobic surfaces.^89^ Research has been conducted based on the use of biocompatible polymers to functionalise CNTs and combine them with other biomolecules to enhance their cell penetration and reduce toxicity, and CPPs serve as a wonderful option.^90^ Xidong et al.^89^ non‐covalently functionalised multi‐walled CNTs (MWCNTs) with low molecular weight chitosan conjugated with TAT, whose results showed that MWCNTs‐TC (TAT‐chitosan‐conjugated MWCNTs) had low cytotoxicity and good water solubility. Its targeted delivery capacity to human breast cancer cell line MD‐MBA‐231 tumour cells was superior to chitosan‐modified MWCNTs (MWNTs‐CS) in both in vitro and in vivo experiments, demonstrating the great application potential of MWCNTs‐TC in tumour treatment. Houmam Kafa et al.^88^ investigated the ability of ANG (Angiopep‐2)‐targeted chemically functionalised multi‐walled CNTs (f‐MWNTs) to cross the BBB in vitro and in vivo. They discovered that conjugating ANG to f‐MWNT significantly enhanced their transport across the porcine brain endothelial cell monolayer in vitro and in vivo and cerebral uptake of f‐MWNT.^88^ F‐MWNT‐ANG exhibited enhanced uptake in the glioma brain compared to the non‐targeted conjugate, demonstrating the potential of ANG‐conjugated f‐MWNTs for effective drug delivery to brain malignancies.

#### 3.1.5 Other NPs

In addition to the most common NPs mentioned above, several other NPs can also be used to combine with CPPs to enhance their anti‐cancer efficacy. In a study, researchers combined Mn‐doped ZnS NPs (Mn:ZnS NPs) and a CPP to deliver chemotherapeutics, PTX. PTX‐loaded Mn:ZnS NPs with different CPPs (PEN, pVEC and R9) showed improved anti‐cancer efficacy compared with bare PTX. Moreover, they studied the in vivo biological distribution and anti‐cancer effect of the conjugate on a breast cancer xenotransplantation model. The H/E staining result confirmed that R9:Mn:ZnS NPs have the maximum PTX loading capability, especially in an acidic environment, which can lead to higher toxicity against cancer cells.^91^

AgNPs are mainly used for antibacterial and anti‐cancer treatment and to promote wound repair and bone healing, or as vaccine adjuvants, anti‐diabetic agents and biosensor adjuvant therapy.^92^ For the sake of cellular uptake improvement, just like other targeted drugs, AgNPs must be targeted to the tumour site to exploit their anti‐tumour effect potential. Functionalisations with different CPP of AgNPs were developed in MCF‐7 cell lines. AgNPs modified with CPPs were found to reduce the survival rate of MCF‐7 cells in a dose‐dependent manner because the chances of cell–particle interactions after peptide coupling were increased, and higher uptake and subsequent toxicity of AgNPs were realised.^93^

### 3.2 Delivery of biological molecules

#### 3.2.1 Nucleic acid

In most cases, the anionic charge and spontaneous binding to the cation CPPs by electrostatic interaction make CPPs appropriate for NA delivery. CPPs have a variety of functions in CPP/NA complexes, including protecting NAs from nucleases, enhancing cell uptake and targeting specific cells. In addition to the direct complexation of CPPs with NAs, CPPs are also used to target NA‐loaded NPs to specific organs and cells.^94^ Furthermore, CPPs can be delivered to plasmids through four steps: firmly condensing and protecting DNA, targeting specific cell surface receptors, destroying endosome membrane and transporting DNA goods to nucleus.^94^

Cancer is usually triggered by mutations in key genes, and studies have found that NA gene therapy can achieve a satisfying anti‐tumour effect.^95^ Common NA therapies include plasmid DNA (pDNA),^96^ messenger RNA (mRNA), small interfering RNA (siRNA)^97^ and antisense oligonucleotide (AON).^98^ NAs are highly charged anionic macromolecules that can be rapidly eliminated from the fluid circulation. Thus, one of the greatest challenges in gene therapy is to address the issue of specific delivery. Previously, viral vectors were used to deliver NAs, but they have been gradually replaced by CPPs due to inherent immunogenicity, limited ability to deliver macromolecules and high treatment cost.^99^ As non‐viral gene delivery vectors, CPPs are capable of delivering membrane‐impermeable compounds into living cells and show a low immunogenic response with a safer and higher gene loading capacity than viral systems.^96^ Coupling CPPs with NAs solves the poor permeability and stability of NAs while achieving effective intracellular delivery. The following examples are mainly about siRNA and DNA.^100^

##### siRNA

It is known that siRNA is a double‐stranded RNA consisting of 20–25 nucleotides that can be used in distinct areas in biology.^101^ Additionally, siRNA is mainly involved in RNA interference (RNAi) to regulate gene expression and is easily filtered by glomerulus in vivo and excreted through urine with the disadvantages of easy degradation, short half‐life and uncontrollable effective drug concentration. Therefore, it has become a widely accepted method to deliver siRNA into cells via CPPs and improve the limitation of siRNA drug application.

At present, CPPs have been a universally used delivery vehicle for the efficient delivery of siRNA into cells. For example, siRNA‐CPPs conjugates have been linked to thermo‐magnetic double‐responsive liposomes (TML). In vivo experiments have demonstrated that siRNA‐CPPs/TML exhibits superior in vivo targeted delivery, anti‐cancer efficacy and gene‐silencing efficiency in the MCF‐7 xenograft mouse model.^102^ In addition, another study published by Hayashi et al. showed that NPs composed of crotamine and DNA/RNA could be delivered as NAs drugs to in vitro and in vivo systems. Crotamine, prepared by recombination, chemical synthesis and fluorescence labelling, is a polypeptide with 42 residues derived from snake venom. It has been studied that a complex formed by crotamine‐coupled DNA/RNA can be selectively delivered to HCT116 colorectal cancer cells in mice for tumour targeting therapy.^103^ WEE1 is found to be overexpressed on many cancer cells.^104^ A novel siRNA delivery system (RRCPP) was constructed experimentally due to the therapeutic effect of siRNA targeting the WEE1 gene on pulmonary melanoma. The system consists of the CPP (R8), cholesterol, PEG and cyclic RGD that binds to the integrin αvβ3 receptor. The results showed that the RRCPP/si WEE1 complex loaded with WEE1 siRNA drugs could significantly inhibit tumour growth in melanoma cancer models with high stability and feasibility.^105^

Based on the above experimental results, CPPs show a broad prospect as an effective and safe carrier for siRNA targeting to tumours in preclinical studies. Many peptides have been combined with the siRNA to target cancer with great success in animal models.^106^, ^107^, ^108^

##### DNA

CPPs can also help deliver DNA into cells and, in some cases, even into the nucleus. The delivery of macromolecules can facilitate gene transfection and impact gene expressions.^109^ A unique double conjugated molecule (six double‐stranded DNA bundles formed by folding six DNA chains) that can improve cellular uptake and effectively target Ramos cells has been developed.^110^

Another example is the study of a novel amphiphilic peptide consisting of repetitive RALA units, which overcomes biological barriers to transfer genes. The results indicated that RALA made a significant contribution to the systemic delivery of NA therapy. In RALA, the lysine residue was replaced by arginine, and the use of arginine in RALA offered a few significant advantages, such as the bind of pDNA and active delivery of DNA to the nucleus within a minimum of a few milliseconds. Additionally, when the peptide was conjugated with pDNA, the NPs loaded with cationic DNA spontaneously self‐assembled to form a stable self‐assembled NP. The RALA/pDNA NPs complex could protect NA drugs from enzymatic degradation and deliver drugs to target sites, achieving the gene delivery and targeted treatment of human breast cancer.^111^

#### 3.2.2 Delivery of proteins

##### Antibody

Anti‐tumour antibodies binding to CPPs can overcome membrane barriers produced by the antibody size. A few strategies have been developed to successfully transport antibodies to cancer cells with CPPs. The team of Shin designed a therapeutic strategy for colon cancer using anti‐cancer embryonic antigen mAb (CEA mAb)‐TAT‐gelonin. The complex CEA mAb ‐TAT‐gelonin showed higher penetration in vivo and in vitro, so it can transport more antibodies into cancer cells.^112^ In addition, CPP‐3E10 is an antibody that can recognise and physically bind to the N‐terminal of RAD51, which is then isolated in the cytoplasm and prevented from binding to DNA to avoid damage and cancer.^113^ By fusing CPPs with the IgG antibody, Gaston et al. found that a specific set of CPPs, the CPP–antibody compound PEP‐1‐BH or PEPth‐BH, could promote antibody entry into cancer cells by combining an antibody specific to CEA with several different CPPs. However, it is also related to the fusion site of IgG and CPP, so it is imperative to select the correct fusion site on IgG.^114^ In addition, by binding domain Z, a combination of the Fc region of immunoglobulin G (IgG) and a polymer of THE LK sequence, an alpha‐helical CPP has been designed to deliver antibodies to cells at the nano‐molar concentration level. Among them, the IgG/LK domain Z complex penetrates cells through the energy‐dependent endocytosis pathway, and most of the delivered IgG can escape from the endosome into the cytoplasm. Simple yet incredibly successful, this intracellular protein delivery approach for nano‐molar level antibodies represents one of the recent achievements in protein delivery. It is expected to promote the development of anti‐cancer therapies based on intracellular antibody delivery.^115^

##### Fusion protein

Fusion protein is a new type of recombined protein produced by the fusion of immunoglobulin and targeting protein including cytokines, receptors, antigenic peptides and other biologically active functional proteins. And a subgroup of CPP fusion proteins are constructed so that they can deliver tumour associated antigens to APCs to induce tumour‐specific immune responses and improve CTL responses.^116^ In such a case, identification or purification of tumour antigens is not necessary for the application of CPP fusion proteins in various tumour immunotherapies. Andrini and Rebollo et al. combined CPP with the interacting peptide PP2A, an essential modulator of tumour‐involved signalling pathways and a tumour suppressor complex, to synthesise a therapeutic peptide DAPT‐C9H that can penetrate cells, inhibit the interaction of PP2A and caspase‐9 and induce apoptosis.^117^ In addition, they added the explanation of SET. Moreover, the therapeutic peptide was found to effectively inhibit tumour growth, including lung and breast cancer, in various animal models. The group of Rebollo also identified the motif that binds PP2A to SET, which is involved in the initiation and development of cancer cells. Furthermore, SET, a universally famous tumour suppressor, is an oncoprotein that decreases PP2A activity. SET also plays a critical role in promoting the development of therapeutic resistance, so it may be a biomarker that predicts drug sensitivity and a therapeutic target to enhance current anti‐cancer treatments.^118^ The motif was fused with a CPP to synthesise a chimeric protein, which was shown to be effective in inhibiting tumour progression, indicating that it could block the interaction between PP2A and SET.^119^ Recent advances have demonstrated the anti‐tumour activity of this peptide in xenograft models of chronic lymphocytic leukaemia and breast cancer.^117^ In addition, FOXM1 is a transcription factor that controls DNA replication and mitosis in cells and can inhibit its own transcriptional activity. The inhibition of FOXM1 can terminate the proliferation of cancer cells. Therefore, an anti‐cancer drug with FOXM1 protein peptides coupled with CPP can be designed to make therapeutic proteins enter cancer cells and play an anti‐cancer role. Zhang et al. fused a CPP‐R9 with a domain of FOXM1 and expressed the chimeric structure in bacteria successfully, demonstrating that the complex R9‐FOXM1 can inhibit the tumorigenic ability of cancer cells and tumour growth in nude mouse xenograft tumour models.^120^

##### Anti‐cancer peptides

Anti‐cancer peptides (ACPs) are short peptides that can be obtained from natural and modified peptides. Numerous studies have explored their advantages, such as high tissue permeability, low incidence of drug resistance, low production cost and easy modification, which are similar to CPPs.^121^ To maximise their anti‐tumour effects, many studies have combined ACPs with CPPs to make the drug targeted more precisely to kill tumour cells. Before being used as an effective anti‐cancer drug, they were only considered as cationic polypeptides isolated from various organisms.^122^ Compared with conventional chemotherapy drugs, ACPs show better performances in inhibiting the proliferation and migration of tumour cells and tumour blood vessels.^123^ The safety, side effects and effectiveness of carrier peptides that target cancer cells directly and/or by activating immune responses have been widely confirmed in clinical trials.^124^, ^125^

##### Vaccine peptide

Currently, a novel polypeptide‐based vaccine, known as vaccine peptides, has gained popularity among researchers to improve the anti‐cancer effect. CPPs in the vaccine show the ability to induce strong cellular responses and exhibit long memory times, and adjuvants are not necessarily required.

Many tumour vaccines were being measured in clinical trials. Pre‐clinical and clinical studies have shown that using CPPs as a vaccine vector can generate a robust cancer‐specific immune response, particularly against ‘cold tumours’ that are less immunogenic. CPPs improve treatment by activating CD8 and CD4 T cells and inducing an adaptive immune response.^126^ For OVA‐specific CD8 T cell immune responses^127^ and penetratin,^128^ these vaccine peptides based on CPP (Z12) have been shown to induce long‐lasting immune responses.^129^ Furthermore, TAT immunity with OVA or HPV‐E7 fusion has shown to be memory‐induced in tumour re‐attack experiments.^130^

Overexpressed in several malignant tumours (including gastric cancer), LY6K derives peptide LY6K‐177 (RYCNLEGPPI) designed for this target, and the latter can be seen as a promising CPP in immunotherapy. The vaccine peptide can induce specific CD8+ cytotoxic T lymphocyte (CTL) responses and antigen‐specific cellular immune responses and suppress LY6K expression, thus effectively reducing tumour size and volume.^130^ A special CPP, GV1001, a 16‐amino acid peptidase derived from human telomerase reverse transcriptase (hTERT), was reported in 2013.^131^ Compared to other CPPs that pierce cell membranes by electrostatic interaction with proteoglycan, GV1001 can deliver macromolecules into the cell by forming complexes with extracellular heat shock proteins 90 and 70.^131^, ^132^ Its role as an anti‐cancer vaccine peptide has been reported in treating advanced pancreatic cancer,^133^, ^134^ non‐small cell lung cancer,^135^ melanoma^136^ and prostate cancer.^137^ In clinical trials, the complex of Hsps with endogenous tumour antigens was extracted and manufactured as a vaccine to cancer patients.^138^ The ultimate success of cancer vaccination depends on the generation of the tumour‐specific CTL.^139^, ^140^ In preclinical models of HPV16‐induced cervical cancer, it was found that inoculation of the unadjuvanted LALF(32‐51) (an antigen‐associated Limulus polyphemus protein) ‐E7 (an HPV antigen) fusion protein significantly improved the presentation of E7‐derived peptides to T cells in vitro and inhibited tumour growth.^140^ Tumour cells can evade the immune system through various monitoring mechanisms, including negatively regulating T cell function by inhibiting the expression of immune checkpoint ligand and blocking the interaction between ligand and receptor antibodies, such as CTLA4‐Ig G, PD1‐Ig G and PD‐L1‐Ig G that have been shown to inhibit various forms of melanoma and several advanced malignancies.^141^

CPPs suffice for the delivery of proteins. Vaccine peptides can induce a variety of immune responses.^142^ CPP such as P28 can produce a post‐translational increase in p53 in tumour cells by inhibiting its ubiquitination.^143^ Thiol‐reactive arginine‐rich peptide additives can enhance the uptake of protein–CPP conjugates in a non‐endocytic mode.^144^ Peptides can interfere with Ras/Raf pathways,^145^ destroy the SET‐PP2A interaction^118^ and target the sites of interaction to terminate downstream pathways for tumorigenesis.^146^ Based on the above results, the immunotherapy that combines immune checkpoint suppression with CPP‐based tumour vaccines represents a promising future direction.

### 3.3 Delivery of chemotherapeutic compounds

CPPs are positively charged peptides, usually 5–30 amino acids long, which overcome the plasma membrane in a non‐invasive manner by endocytosis or the direct translocation pathway without disrupting the membrane integrity.^147^, ^148^ Traditionally, chemotherapy drugs are used to treat cancer due to their capacity to prevent cancer cells from proliferating, attacking and metastasising. Generally speaking, most chemotherapeutic drugs are short of tumour cell specificity, whereas CPPs can mediate the translocation of conjugated products (such as anti‐cancer drugs) to cross the cell membrane. In addition, the combination of anti‐cancer drugs and CPPs can increase the cell membrane permeability, drug delivery, drug half‐life and accumulation in tumour cells.^149^

Next, based on the combination of CPPs and various anti‐tumour drugs, we will discuss the targeted inhibition effects on different tumour cells. Five anti‐tumour drugs are natural anti‐tumour drugs, antibiotic antineoplastic drugs, platinum chemotherapeutic drugs, antimetabolite antineoplastic drugs and hormone anti‐tumour drugs.

#### 3.3.1 Natural anti‐tumour compounds

##### Epigallocatechin gallate

Epigallocatechin gallate (EGCG), originally derived from green tea, has an anti‐cancer effect. The design of DDSs was based on three components: EGCG (anti‐cancer drug), colloidal mesoporous silicon dioxide (CMS) (drug carrier) and PEGA‐pVEC peptides. The in vivo experiment and HE staining were perfected to confirm that it had the effect of targeted therapy for breast cancer. Moreover, the coupling of EGCG with CMS and PEGA‐pVEC peptides enhanced its own anti‐cancer effects according to the results of CCK‐8 detection, confocal imaging, cell cycle analysis and western blot.^11^

##### Camptothecin

As a natural product, Camptothecin (CPT) is derived from Camptotheca acuminata and exhibits anti‐cancer activity by inhibiting topoisomerase I, which causes DNA damage.^150^ Although CPT has significant tumour inhibition ability, it is too hydrophobic to be soluble in water, which limits its further clinical application.^151^ By designing a novel pH‐activatable CPP, LHHLLHHLHHLLHH‐NH 2(LH), the conjugate showed more significant pH‐dependent anti‐cancer activity than free CPT after binding CPT to LH.^152^

##### Curcumin

Curcumin (CUR), a component of the turmeric plant, can make dishes yellow and be used as spices. As a natural substance, CUR shows anti‐inflammatory and anti‐oxidant effects but low toxicity in overdose.^153^ The anti‐tumour property of CUR has also been confirmed on some tumours.^154^ A CPP‐mediated CUR nano‐micelle was proposed for the efficient accumulation of chemotherapeutic drugs in glioblastoma multiforme(GBM) tumour cells. Extensive researches have demonstrated the multi‐targeting ability of CUR to tumour cells and the low toxicity to normal cells. Moreover, it has also been speculated to inhibit MDR‐related proteins.^155^ In another recent study, the tumour‐targeting mechanism and the effect of the cellular uptake of MMP‐reactive CUR NPs on the breast cancer cell line MCF‐7 were analysed in vivo and in vitro, respectively. In vivo pharmacokinetics demonstrated that CUR‐P‐NPs are more targeted to MCF‐7 xenografts than normal tissues, causing significant increase in bioavailability.^156^

##### Paclitaxel

Based on the advantage that most CPPs are hydrophilic, the water solubility of PTX‐CPP is developed, thereby avoiding the consequences of using inorganic solvents to reduce the drug efficacy. The conjugates, PTX‐TAT and PTX‐LMWP, were designed to address the poor resistance and solubility of PTX. PTX‐CPPs were proven to enhance cellular uptake and induce apoptosis in A549 and A549T cells compared to free PTX. The analysis of cell cycle distribution showed that PTX‐LMWP inhibited the mitosis of drug‐resistant A549T tumour cells by a different mechanism from PTX, further proving that PTX‐CPP was more effective than free PTX in inhibiting the growth of tumour‐bearing mice.^157^

In addition, a novel CPP‐RRRRRw (R7) was designed to inhibit tumour growth by accelerating the translocation of PTX to HeLa cells. In vivo animal experiments with tail vein injection of the R7/PTX complex showed significant tumour inhibition.^158^ However, due to the instability of the non‐covalent bond between R7 and PTX, heparin caused the dissociation of the R7‐PTX complex. Therefore, they constructed a novel CPP 9 (rrrrrrrwpp), which has the same hydrophilic and modified hydrophobic moieties as R7. In vivo experiments showed that the intravenous P9‐PTX complex significantly inhibited tumour growth and was not easily degraded. In addition, almost all cells survived even at 1 mM, indicating that P9 was non‐toxic at physiological pH. This latest study was based on the CPP(R7) coupling experiment with PTX.^159^

##### Docetaxel

Docetaxel (DTX), similar to PTX, belongs to the family of toxoids.^160^ DTX has been confirmed to show inhibitory activity on breast cancer, NSCLC, and other tumours.^161^ Cyclodextrin can be used to form a complex to increase the solubility and bioavailability of DTX. A novel DDS—D‐CNP (DTX‐cyclodextrin‐polylactic acid‐glycolic acid (PLGA) NPs)‐R7 synthesised by a double emulsification method can remarkably improve the absorption and bioavailability of DTX as well as its cellular uptake, which is verified by its excellent treatment efficacy on breast cancer cells.^162^

#### 3.3.2 Antibiotic antineoplastic drugs

##### Doxorubicin

As an anti‐cancer drug for treating solid tumours, small molecule DOX has cardiotoxicity, and some tumours may be DOX‐resistant, limiting its further clinical application. Compared with the single drug DOX, the combination of CPPs and DOX (CPP‐DOX) has been proved to possess better therapeutic effects and lower cytotoxicity.^163^ In addition, anti‐DOX properties found in human breast cancer cell lines do not affect tumour cell apoptosis induced by the CPP‐DOX complex.^164^

Thermosensitive liposomes (TSLs) consisting of a heat‐activated CPP‐DOX conjugate have been constructed, taking asparagine‐glycine‐arginine (NGR) peptides as a target to improve the specific therapeutic effect of tumours. The CPP‐DOX conjugate in TSL is designed to mask and protect CPPs. In vitro experiments of HT‐1080 and MCF‐7 cell lines, researchers verified the specific targeting ability of the liposome to HT‐1080 cells and enhanced the drug delivery ability of tumour cells.^164^, ^165^

A CPP–KRP rich in lysine is different from common CPPs used as drug carriers. Moreover, KRP is a polypeptide composed of 54 amino acids that can achieve long‐term inhibition in solid tumours. Through stable connection with DOX, KRP‐DOX has multiple synergistic functions, one of which is the protection of normal tissues from the toxicity of DOX.^165^ Therefore, KRP is used to enhance penetration and retention.^166^, ^167^ In addition, targeted to osteosarcoma (MG63) with specificity and stability, the KRP‐DOX conjugate hardly releases free DOX in blood circulation, thus avoiding toxicity to normal tissues.^273^

##### Clotrimazole

Experimental groups have reported the synthesis of novel CPP2‐thiazole conjugates that successfully bond to PTX and clotrimazole (CLZ) as well as produce enhanced anti‐cancer effects and synergistic inhibition of cancer cell proliferation, mainly in PC‐3 cells. Interactions with drugs can be enhanced by peptides with osmotic properties. As cell lines are distinct in metabolism with specific characteristics, more research should be conducted on other cell lines such as breast and lung cancers. Further studies in animal models and clinical trials are highly requested to confirm these preclinical results.^168^

#### 3.3.3 Platinum chemotherapeutic drugs

##### Oxaliplatin

Platinum‐based drugs are one of the main active drugs to treat colorectal cancer. A CPP―R8‐oxaliplatin conjugate enables the rapid and successful delivery of oxaliplatin to colon cancer cells. Moreover, the conjugate can effectively inhibit tumour growth and showed relatively high tumour target ability in vivo.^169^

#### 3.3.4 Antimetabolites

Methotrexate (MTX) is a high‐dose chemotherapy drug used to treat malignant tumours in children and adults, such as acute lymphoblastic leukaemia, osteosarcoma, lymphoma, lung cancer and breast cancer.^170^ However, MTX is unsatisfactory in tumour targeting ability and stability and might exert toxicity on normal organs and tissues of the body. Additionally, a certain degree of drug resistance has been reported.^171^ The selective CPP (RLWMRWYSPRTRAYGC)‐functionalised polymer (SCPP‐PS) was found to mediate efficient and targeted delivery of MTX to human lung cancer cells in vivo. MTX‐SCPP‐PS showed much lower IC values and the slowest tumour growth rate than the free groups, MTX and MTX‐PS.^172^ Two different CPPs, YTA2 and YTA4, were conjugated to MTX. The two conjugates were quantified by the fluorescence quantitative method. At a concentration of 1 mM, the peptide‐MTX conjugates were shown to overcome MTX resistance and kill cells more efficiently than MTX alone. It was found that the DDS increased the targeting ability of breast cancer and solved the problem of drug resistance to a certain extent.^173^

#### 3.3.5 Hormone therapy agents

As a widely used oral hormone anti‐tumour drug, tamoxifen (TAM) can be used to treat ER‐positive breast cancer,^174^ ovarian cancer^175^ and so on. Researchers have investigated anti‐tumour activities of Ridaifens (RIDs), which are a series of synthesised TAM derivatives. It was reported that (RID)‐A,^176^ ‐B, ‐D and ‐F could inhibit the protease activity of the 20S proteasome by detecting the proteasome inhibition of TAM derivatives. Among them, the inhibition of the RID‐F proteasome was the strongest. The TAM derivative RID‐F inhibits the human 20S proteasome,^177^ but it showed poor inhibition on the 26S proteasome. Various RID‐F peptide conjugates have been prepared experimentally to overcome their drawbacks. Firstly, the peptide fragment was recognised as a substrate, thereby facilitating the entry of the conjugate into the catalytic chamber. Secondly, the polypeptide fragment promoted penetration of the conjugate into cells and increased intracellular RID‐F concentration to a level sufficient to inhibit the proteasome. The results denoted that the R8‐containing conjugate of RID‐F inhibited the 26S proteasome and intracellular proteasome in KMS‐11 cells.^178^ Reports have evidenced that the use of CPP for the delivery of chemotherapy may be therapeutically valuable due to enhanced pharmaceutical features.

In conclusion, CPPs can transport exogenous molecules across the plasma membrane barrier to target sites. CPPs firstly bind to the plasma membrane through electrostatic interaction, and then induce membrane instability or reversed micelles in the lipid bilayer to achieve uptake of CPPs. CPPs are usually used to transport different kinds of goods due to their high permeability. Coupling CPP with chemotherapeutic drugs such as SynB1 delivers paclitaxel to breast cancer cells to induce tumour cell cycle arrest and tumour cell apoptosis,^179^ TAT and LMW deliver paclitaxel to drug‐resistant lung cancer to affect tumour cell mitosis and inhibit tumour growth^180^ and that D‐MCa can deliver cisplatin to human glioblastoma cell to trigger the ROS‐ERK/AKT‐p53 pathway to induce apoptosis.^181^ However, some CPPs still have intrinsic activity on tumour cells, just as P28, which can combine with wild‐type and mutant p53 to exert anti‐cancer effect. As mentioned above, the role of CPPs in penetrating the cell membrane and delivering therapeutic molecules to specific intracellular sites is proved to be robust against different cancers. Despite the overall satisfying results reviewed here, few treatments achieve the expected clinical efficacy. Related limitations should be settled urgently to achieve satisfactory clinical therapeutic efficacy.

## 4 OPTIMISATION STRATEGIES OF CPPs

### 4.1 Increasing stability

CPPs can function as molecular drug transporters to effectively promote the carrying substances crossing the physiological barrier. The function of CPPs depends on physical and chemical integrity and stability. However, traditional CPPs have a short half‐life in vivo, mainly due to their impaired resistance to proteases and their inability to escape from the endosome. On this basis, a new generation of peptides with optimised properties has been continuously developed.^182^

There is a need to develop CPPs with the better penetrating ability and higher stability. At present, the stability of CPPs can be improved by different methods. When their structure is changed, they cannot be recognised by proteases or be degraded.

#### 4.1.1 Chemical modification

The degradation of CPPs is mainly caused by proteases. Therefore, CPPs can be chemically modified to improve stability, including terminal improvements, employment of unnatural amino acids, side‐chain modifications, peptide backbone modifications, chiral changes and enzyme cleavage site modifications. In this way, the recognition by proteases can be avoided.

N‐terminal acetyl capping (deacetylation) and C‐terminal amidation were used to improve the stability of exopeptidase degradation. As the tetrapeptide Arg‐Leu‐Tyr‐Glu(RLYE) can be degraded by aminopeptidases B and N, its N‐terminal deacetylation can extend the half‐life of the tetrapeptide from 1.2 to 8.8 h.^183^ The conversion of natural peptides to synthetic peptides increases the resistance to cleavage. The most representative example of non‐natural amino acids is α‐aminoisobutyric acid (Aib), which improves peptide stability through artificial intervention (e.g. α‐helix restriction on the conformational freedom of the opposite side chain).^184^ A fluorine thiol displacement reaction (FTDR), which renders a class of peptide analogues, especially the FTDR‐stapled lead Axin and p53 peptide analogues demonstrated enhanced inhibition of tumour cells.^27^ As the peptide bond is the true hydrolytic substrate, the peptide can be protected from proteolytic degradation by modifying the structure of the peptide bond, such as N‐methylation and N‐alkylation. Another common chemical modification strategy is using D‐amino acid instead of its l‐amino acid counterparts because of its protease resistance, which has been utilised to enhance the stability of CPPs, such as TAT, R9, osmotic protein, hLF, pVEC and sweet arrow peptide. The in vivo half‐life of D‐peptide is longer than that of L‐peptide, and D‐polyacrylate CPP has been successfully designed as a cancer contrast agent.^185^ Thus, it is feasible to use D‐isomers to substitute L‐amino acids to increase the half‐life for overcoming protease susceptibility.^186^ In addition, there is an enzyme cleavage site for peptide degradation in vivo, which can be modified in different ways to avoid degradation by proteases and enhance metabolic stability.^187^, ^188^ Metabolic study of Tat peptide has shown that the primary initial degradation fragment occurs at the C‐terminal Arg residue.^188^ Therefore, substituting this Arg residue with Lys, Asn or Ala may help to improve peptide stability and without altering penetration.^189^ In addition, CPPs can be coupled to polymers to protect their integrity and stability. Polyethylene glycol (PEG) is a polymer, and the half‐life of Tat conjugated with PEG and phosphatidylethanolamine is three times higher than that of unconjugated peptide.^182^ Moreover, the CPP‐coupled NPs can also resist the hydrolysis of protease and protect themselves from degradation. PH‐sensitive PEG liposomes are modified by TAT and target‐specific part (mAb 2C5), allowing specific injection of chemotherapeutic agents into tumour cells.^190^ In addition, the determination of specific protease cleavage sites can improve the stability of CPP. The use of polysaccharides as the outer shell can protect the protease cleavage site of the cell‐penetrating polypeptide complex, thus greatly improving the stability.^185^

#### 4.1.2 Conformational change

Conformational changes can improve the structural stability of CPPs and protect peptides from protease degradation, including backbone cyclisation, disulphide and conformational freezing. At present, when short peptides rich in arginine and hydrophobic residues are embedded in small and medium cyclic peptides (7–13 amino acids), backbone cyclisation can be applied to arginine‐rich CPPs that are bound to the cell membrane. According to confocal microscope analysis, the chimeric peptide was found to have higher cellular permeability and stability than linear low‐arginine peptides, with two to five times higher permeation efficiency than the original peptides.^191^ Construction of intramolecular disulfide bonds in the therapeutic peptide GLP‐1, a cyclic conformation mimetic (TY70517), extended the half‐life from 2 min to 52 h.^192^ Disulfide induction enhances thermodynamic stability and improves the endosome escape, largely stabilising the peptide structure.^193^ Conformational freezing can be achieved by covalent cross‐linking between amino acid side chains.

In summary, focusing on the synthesis of CPP sequences, the optimisation of CPP sequences and the modification of CPP structure is important to improve the stability of CPP. Good stability can be ultimately achieved, ensuring the drug delivery to the target cellular components in complex with CPP.

### 4.2 Increasing specificity

**FIGURE 4:**
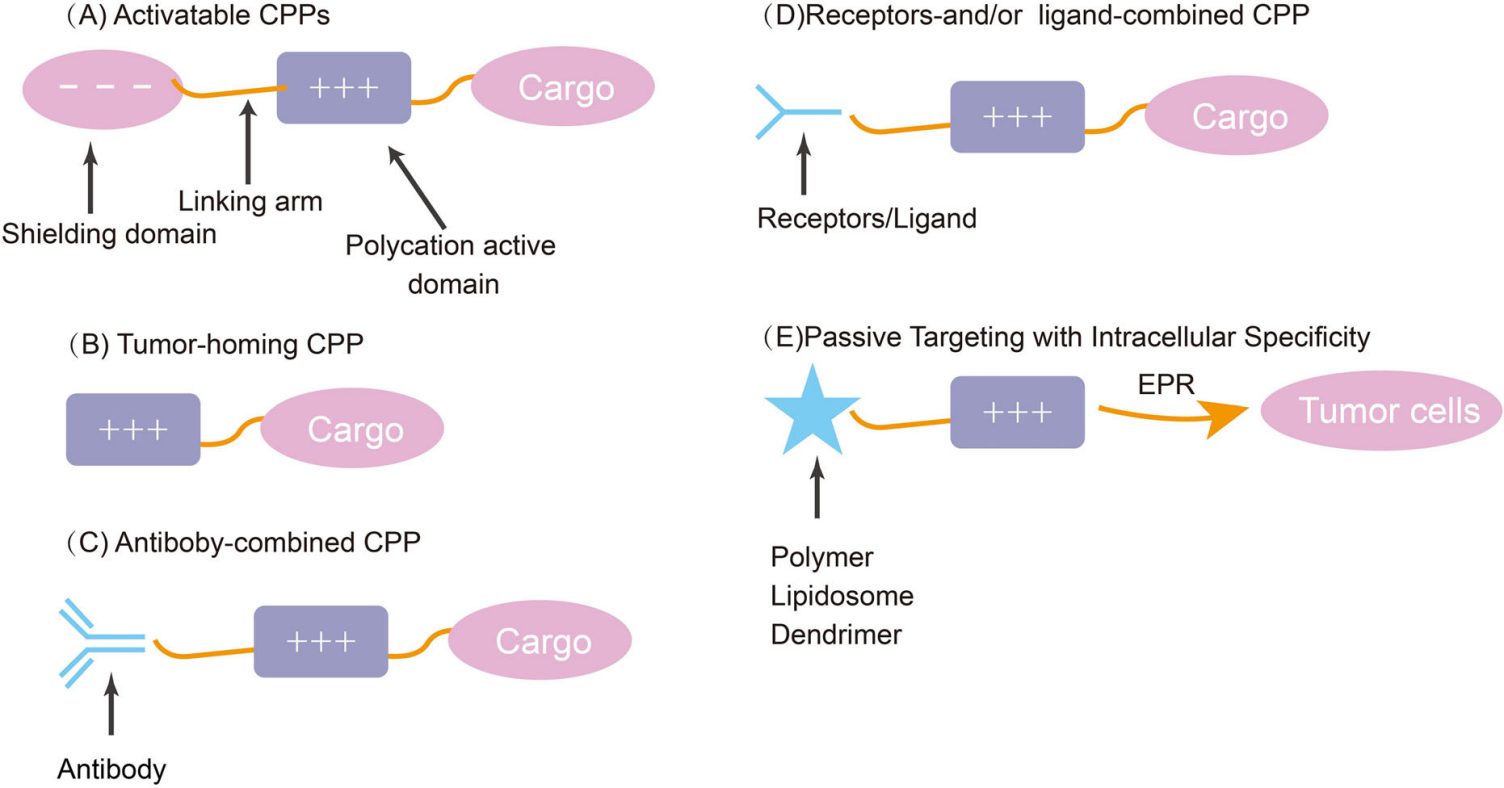
Mechanisms to enhance the specialty of CPPs. Several schematic diagrams of CPP compounds to improve the specificity: (A) The polycation active domain is cationic and the shielding domain is anionic. The cleavable linking arm is the key to specificity. (B) The cationic peptide allows selective entry of the cargoes into cells. (C) The combination of CPPs with the antibody has been proved to improve the specificity. (D) Works by targeting receptors or ligands. (E) EPR refers to the enhanced permeability and retention effect. Passive targeting based on EPR can be utilised to improve the specificity of CPPs

The specific targeting ability and good cell penetration are two important characteristics that must be possessed by the tumour drug‐targeted delivery system.^194^ To improve the specificity of CPPs, many researchers have proposed improvement schemes. In addition to improving specificity, some methods can also improve other defects of CPPs, such as the endosome escape rate. In 2004, Tsien's group proposed the concept of activatable CPPs.^195^ Based on this idea, a series of similar targeted delivery systems can be designed and developed to control the activity of CPPs by introducing environment‐sensitive ‘switches’. Another conventional strategy is to introduce certain tumour cell‐targeting ligands, ranging from sugars to peptide sequences and even full‐size antibodies. These ligands can bind to CPPs biologically or chemically, making them eligible to design targeted transport systems for tumour cells. In the following sections, some popular and effective strategies from current studies are presented in detail for improving the tumour targeting of CPPs, as shown in Figure 4.

#### 4.2.1 Activatable CPPs

**FIGURE 5:**
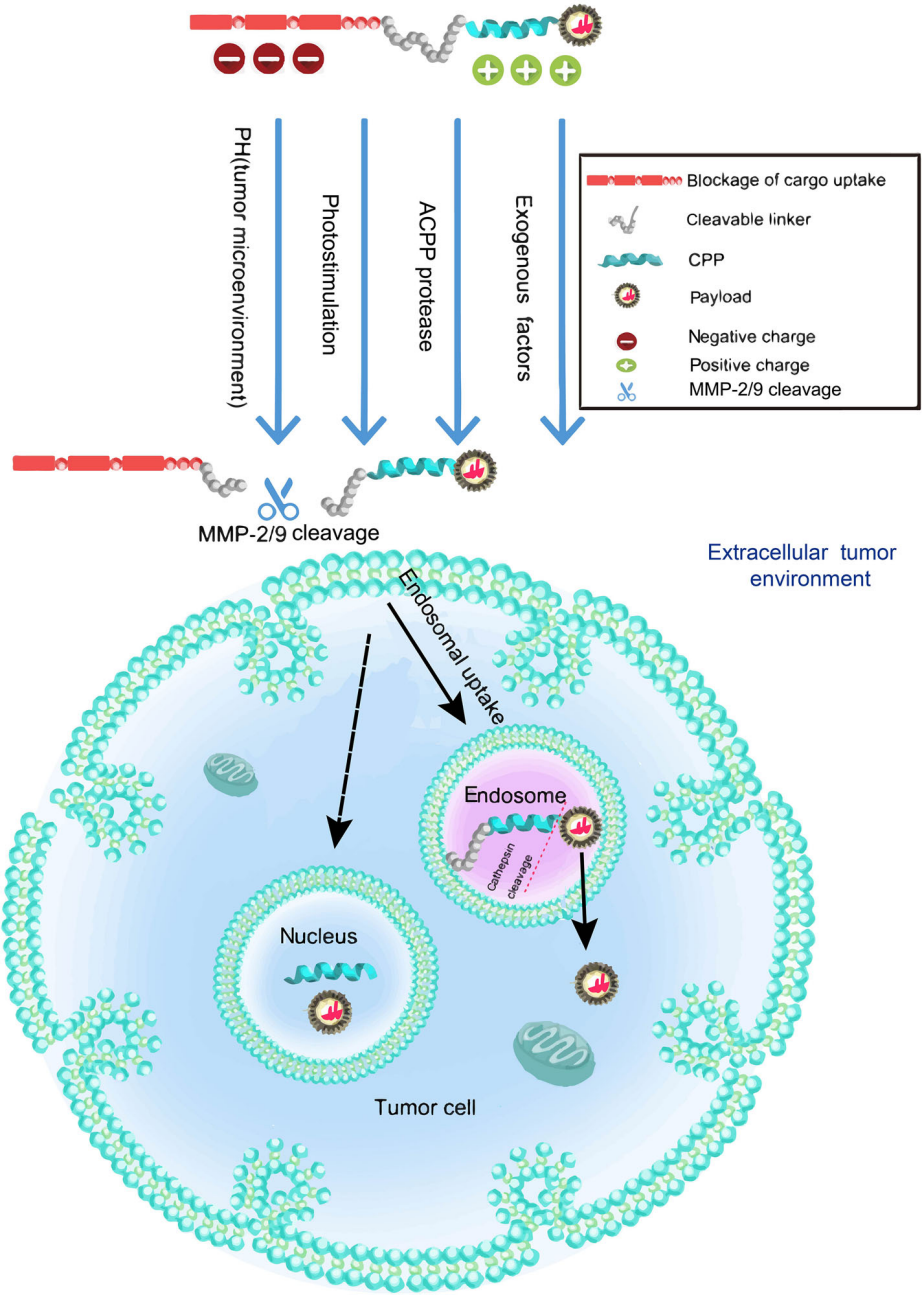
The activation and cellular uptake of ACPPs. ACPPs are a new type of carrier that can be activated by special enzymes in the tumour tissue site to induce cell penetration. The molecular structure generally includes three functional regions: polycation active domain with the cell‐penetrating ability (e.g. CPPs); a cleavable connecting arm; polyanion shielding domain. As shown in the figure, there is the high‐expressed protease at the tumour site. Certain internal environmental factors such as low pH, and external physicochemical stimulation such as light and exogenous substances are the conditions that can dependently trigger cleavage between polycation active domain and polyanion shielding domain. This schematic diagram selects the most potential protease MMP2/9 as a shear to activate the activatable CPP compound. After cleavage in specific sites, they can enter tumour cells by uptake mechanisms. The dashed arrow means the entrance into the nucleus is sometimes observed, and the mechanism is not fully understood

The first proposed activated CPPs (ACPPs) are novel carriers that can be activated by special enzymes at tumour tissue sites to induce cell penetration. The molecular structure generally included three functional regions: polycation active domain with the cell‐penetrating ability (e.g. CPPs), a cleavable connecting arm and a polyanionic shielding domain.^196^ In particular, the electrostatic interaction between the polycationic part and the polyanionic part temporarily inhibits the cell penetration of the polycationic part, whereas the connecting part between them can be broken upon stimulation by factors such as protease. It solves the problem of insufficient tumour targeting and masks cations in the serum, which prolongs drug circulation.^196^ The dependent cleavage triggering conditions include highly expressed proteases at the tumour site, internal environmental factors such as low pH and external physicochemical stimulation such as light and exogenous substances.^196^ All these descriptions can be seen in Figure 5.

In 2009, Nobel laureate Tsien's team developed the first ACPP protease, targeting multiple xenograft tumour models at different cancer sites and transgenic models of spontaneous breast cancer. Membrane‐bound and secreted proteases (mainly matrix metalloproteinases) of cancer cells can cleave the junction between the polycationic CPP and the polyanionic neutralising agent, thereby activating CPP. After activation, the released peptides can transport cargoes into cancer cells, thereby improving cell selectivity. To further evaluate the applicability of ACPPs to cancer types, the team studied subcutaneous fibrosarcoma, melanoma and various xenografts models, including cervical, prostate, colon and breast cancer. The results showed that compared to the negative control group (type D), ACPPs consisting of L‐type amino acid substrate peptides exhibited significant distribution selectivity with a 2‐fold to 6‐fold increase in their accumulation at the tumour site.^197^, ^198^

Due to the heterogeneity of protease expression levels and intratumoral pH,^199^ the above‐mentioned ACPPs sensitive to the tumour microenvironment may not be effectively activated in some patients. External stimuli like light are certainly an ideal choice. This factor is unaffected by the extracellular conditions of the tumour microenvironment, and the chemical bonds of the photoreactivity can be cleaved or isomerised at specific times and locations, thus activating CPPs on demand.^196^ Xie et al.^200^ developed a liposomal delivery method based on a photo‐responsive peptide and an NGR peptide. The positive charges of the lysine residues on CPPs were temporarily locked, which can be unlocked via near‐infrared (NIR) light to form photo‐sensitive peptides (PSPs). The findings showed that the suggested PSP/NGR‐L improved cancer cell recognition and selective uptake. In a nude mice with HT‐1080 tumour, greater anti‐tumour effectiveness was established under NIR illumination.

CPP Pd‐TAT can regulate tumour cell migration under mild laser irradiation and improve photothermal therapeutic performance.^201^ In addition to the cleavage functions of proteases and the microenvironmental specificity of tumour tissues, the intervention of other exogenous factors can lead to the dissociation of the anionic domain from the cationic CPPs. Huang et al.^202^ developed a protein complex consisting of two parts: an antibody targeting part bound to molecular heparin (Hep); a part of the moiety connecting Asparaginase (ASNase) and TAT peptide by disulfide bonds. After reaching the target site, the exogenously introduced protamine sulphate binds more tightly to heparin, displacing the TAT drug moiety from the heparin antibody complex.

#### 4.2.2 Tumour‐homing CPPs

The most direct way to improve the cellular specificity of CPPs is to develop corresponding tumour‐homing CPPs. Tumour‐homing CPPs are oligopeptides that consist of 30 or fewer amino acids and can effectively and specifically bind to tumour cells. It has potential applications in establishing a new non‐invasive tumour‐imaging system for diagnostic and therapeutic purposes. Tumour‐homing peptides like iRGD have three independent modules, namely a vascular homing motif, an R/KXXR/K tissue penetration motif and a protease recognition site. These three parts cooperate together to achieve tumour targeting. Among them, the second sequence motif, R/KXXR/K, is more critical in the tumour‐penetrating properties of iRGD. In addition, it is only when the second basic residue is exposed at the C‐terminus of the peptide that this C‐end Rule (or CendR) motif can be active.^203^, ^204^ More details can be seen in reference.^204^ And to put it another way, unlike ab‐targeting, the targeting effect of tumour‐homing peptides belongs to ligand‐receptor targeting with higher bioactivity and applicability.^205^ So far, many tumour‐homing CPPs have been put into research (as shown in Table 3).

**TABLE 3:** Some examples of tumour‐homing peptides

Peptide	Sequence	Cargo	Cancers	Refs.
Angiopep‐2	TFFYGGSRGKRNNFKTEEY	TAT‐PAPTP	Glioblastoma	^293^
CREKA	CKDEPQRRSARLSAKPAPPKPEPKPKKAPAKK	F3	Triple negative breast cancer	^294^
gHo	–	pVEC	Glioma	^295^
iRGD	CRGDK/RGPD/EC	ST‐4	Breast cancer melanoma	^296^
PL3	AGRGRLVR	AgNPs	Glioblastoma prostatic cancer	^297^
TT1	AKRGARSTA	NPs	Breast cancer	^298^
iNGR	–	PEG‐PTX8	Triple negative breast cancer	^299^
LyP‐1	–	–	Breast cancer	^300^

One of the most common methods for identifying tumour‐homing CPPs is biopanning, using a peptide library based on in vivo phage display technology.^206^ Phage display technology has been widely used in the development of tumour‐targeting drugs. RGD peptide is commonly employed as a specific ligand for the integrin αvβ3 family, which is overexpressed on many malignant cancer cells. The binding of them can induce endocytosis and extracellular matrix remodeling.^207^, ^208^ Vascular endothelin‐2 (Angiopep‐2, ANG) peptide from the aprotinin Kunitz domain can specifically target brain cells and bind to low‐density lipoprotein receptor‐associated protein‐1 (LRP1) to achieve targeted delivery, which is overexpressed in the BBB and glioma.^209^, ^210^, ^211^ IRGD (CRGDKGPDC) binds to αvβ3 integrin, which is encapsulated by neuropilin 1 (NRP 1) after its cleavage, and then enters tumour cells through endocytosis. Due to the specificity of these two receptors, iRGD can target multiple tumour cells.^212^ PL3 can be taken in by glioblastoma and prostate cancer cells through endocytosis by binding to Tenascin C and NRP 1, thereby improving the targeting specificity of the delivery system.^209^ A tumour‐targeted CPP with high efficiency derived from heparin‐binding domain (HBD) of Midkine (named HMD) was discovered in 2022. HMD exhibited higher delivery efficiency than classic CPPs (Tat and R9) and manifested selectivity in tumour cells.^213^ Another strategy for targeting tumours is coupling CPPs with tumour‐homing domains (e.g. pVEC binding to PEGA homing domains). The homing domain is ordinarily impermeable to the cell membrane, but its attachment to CPPs allows effective and selective entry of drugs into the tumour.^39^ Myrberg et al.^214^ conjugated the PEGA homing domain that was previously shown to be capable of accumulating in breast tumour vessels to pVEC. In vitro experiments showed that the PEGA–pVEC complex had a strong tumour targeting ability, and the complex mainly accumulated in the blood vessels of breast tumour tissues. Moreover, when the anti‐cancer drug chlorambucil was conjugated to PEGA‐pVEC chimeric peptide, its anti‐tumour efficacy was increased by more than 4‐fold compared with that of the anti‐cancer drug alone.

#### 4.2.3 Antibody‐combined CPP delivery

In addition to the homing substances already described above, there are many other homing substances such as antibodies or ligands (peptides, oligonucleotides, sugars or vitamins). They can be coupled with CPP to achieve tumour cell targeting specificity through antigens or receptors on the tumour cells’ surface. The carried drugs can also be targeted to tumour tissues by these homing substances to enable targeted therapy. Ye et al.^215^ developed a system called ‘antibody‐targeted triggered electrically modified pro‐drug type strategy’ (ATTEMPTS). The ATTEMPTS system consists of an antibody linked to heparin and a drug component modified by a CPP. Once the complex accumulates at the targeted site, the CPP‐cargoes will be triggered by the subsequent release of the second trigger. The complex remains inactive in the humoral circulation of the targeted tumour to avoid its toxic effects on normal tissue cells.

Researchers have combined a CPP TAT‐Mu (TM) and a targeting ligand HER2 antibody mimetic‐affibody (AF) to design a complex TMAF that can deliver NAs to cells. The effectiveness of TMAF protein to transfect DNA has been confirmed in a xenotransplantation mouse model. It can be seen that the AF‐based chimeric polypeptide with the property of binding to NAs can hint an effective tumour‐specific strategy for the delivery of therapeutic NAs.^216^ Shin et al.^112^ used a non‐internalised CEA mAb to design a novel therapeutic strategy based on tumour targeting using a non‐internalised CEA mAb. This strategy exploited the property of CPPs to deliver an extremely effective and cell‐penetrating protein toxin gelatin peptide intracellularly. In a preliminary in vivo study using LS174T s.c. xenografts in tumour‐bearing mice, TAT‐gelonin/T84.66‐Hep was selectively and significantly delivered to tumour target sites, with a 58‐fold increase compared to TAT‐gelonin alone. In addition, significant tumour growth inhibition (46%) and the absence of gelonin‐induced systemic toxicity were observed.

#### 4.2.4 Receptors/ligand‐combined delivery

Compared with many other normal cells, another manifestation of the special microenvironment of tumour tissues is the presence of some overexpressed receptors and enzymes on the surface of tumour cells,^217^ which can also be used for the targeted design of CPP‐based DDS. The folate receptor is highly expressed in various cancers, including endometrial cancer, renal cell carcinoma, lung adenocarcinoma, mesothelioma and some breast cancers, and can be regarded as a therapeutic target region.^218^ Kang et al.^208^ designed a new double ligand‐modified liposome, which is a liposome nano‐carrier (FP‐Lipo) conjugated with folate and Pep‐1 peptide. The experiment results showed that the dual ligand‐modified liposomes have a higher tumour targeting delivery capacity, providing ideas for treating folate receptor‐positive tumours such as certain lung cancers and bladder cancers.^219^ Similarly, integrin αvβ3, which is abundant on many tumour cells, is able to recognise the RGD sequence in native ligands. Liu et al.^220^ coupled a specific cyclic RGD peptide ligand with CPP‐R8 to design a multifunctional peptide R8‐RGD. In contrast to using R8 and RGD alone, the cellular uptake of R8‐RGD complex liposome was increased significantly. R8‐RGD, a multifunctional peptide, efficiently penetrated three‐dimensional glioma spheroids and the BBB in vitro. During in vivo experiments, R8‐RGD‐lipids were efficiently transported to the brain and selectively accumulated in glioma foci in C6 glioma mice following systemic injection.

Some researchers also employed two strategies to achieve tumour‐targeted delivery simultaneously for better anti‐tumour effects. The epidermal growth factor receptor (EGFR) is another valuable target site for tumours. In the previous study, researchers designed a conjugate of synthetic CPP (corresponding to amino acids 38–59 of human lactoferrin) and the recombinant llama single‐domain antibody (VHH) 7D12, which can bind to human EGFR and is generated through sortase A‐mediated transpeptidation. The complex blocked EGF‐mediated activation of EGFR and inhibited tumours more efficiently than when the drug was used alone, whereas the binding between VHH and EGFR endowed the tumour selectivity of the conjugate.^221^ In addition, Lu et al.^222^ constructed a tandem peptide TAT‐AT7 and tested its ability to bind to VEGFR‐2 and NRP‐1 to target blood vessels and cross the BBB. They synthesised TAT‐AT7‐modified PEI polymer (PPTA) and prepared pVAXI‐En‐loaded PPTA nano‐composites (PPTA/pVAXI‐En). The results showed that PPTA/pVAXI‐En significantly inhibited the formation and migration of vascular endothelial cells, inhibited the growth of gliomas, and reduced the number of microvessels in nude mice with U87 glioma in situ.

#### 4.2.5 Passive targeting with intracellular specificity

The EPR effect, also known as passive tumour targeting, is a phenomenon achieved by taking advantage of the anatomical features of tumour tissue (including large vascular endothelial cell gap, lack of smooth muscle layer of the vascular wall and the reduction of lymphocytes at the tumour site affecting lymphatic return).^223^ These characteristics improve the cell permeability of macromolecular therapeutic drugs in tumours, prolonging their residence time in tumour tissue sites. On the contrary, the normal tissues do not have these structural characteristics, which means that anti‐tumour strategies based on these anatomical characteristics can shield the toxic effects of drugs.

This effect can improve the specificity of anti‐tumour drugs in tumour imaging or treatment with combination systems of CPPs and NPs.^224^ HPMA is a copolymer that specifically targets tumour tissue by a passive targeting process (EPR effect). Xiang et al.^225^ designed a new tumour‐targeting DDS by linking dNP2 to HPMA. The human‐derived CPP‐dNP2 can promote more specific uptake of anti‐cancer drugs in tumour cells and improve anti‐cancer effects. More importantly, dNP2 is less toxic than the traditional CPP‐R8. The results indicated that DDSs based on dNP2 have good prospects for clinical anti‐cancer treatment. In addition, some researchers have utilised the hypoxia characteristics that are common in most tumours. Hypoxia‐inducible factor (HIF) is the main effector of the cellular response to hypoxia, promoting the survival and development of cancer cells. Moreover, overexpression of ETD (ERK targeting domain) variants can result in the inactivation of HIF‐1.^226^, ^227^ In order to directly introduce these ETD forms into cancer cells, Karagiota et al.^228^ designed a CPPP by fusing ETD with the HIV TAT‐ sequence. The results showed that the transduced nuclear TAT‐ETD peptides limited the migration of cancer cells grown under hypoxic conditions and triggered the death of tumour cells, whereas they did not work in cells with normal oxygen content.

### 4.3 Increasing efficiency

In the current application of CPPs, such as intracellular delivery of peptides and proteins, NAs, chemotherapeutic drugs and NPs, endosomal escape is a non‐negligible problem. However, these processes are usually achieved by endocytosis and are trapped in endosomes.^229^ In the absence of endosomal escape, the ultimate result is degradation. Only the cargoes that escape the endosome can enter the cytoplasm and reach the cytoplasm or nucleus, exhibiting a variety of biological activities and exerting the corresponding therapeutic effects. Biomolecules trapped in the endosomes cannot exert a therapeutic effect because they cannot reach the target sites in cytoplasmic.^230^ In addition, CPP‐cargoes are degraded by acidic environments or hydrolases when entering into advanced endosomes or lysosomes.^231^ In 2022, a tricyclic TAT compound can deliver functional IgG antibodies and Fab fragments and overcome endosome to inhabit proliferation of tumour cells.^232^

A study in 2016 showed that endosomal escape domain (EED) plays an important role in endobody escape, which significantly enhances cytoplasmic transfer efficiency without cytotoxicity.^233^ Several researchers combined VH of EpCT65‐AAA with VL of EpCT05 to synthesise EpCT65 with endosomal escape ability, which has shown tumour cell‐specific cytoplasmic penetration activity. EpCT65 can be effectively localised in the cytoplasm of only EpCAM‐expressing tumour cells, with an approximately 2‐fold increase in endosomal escape ability compared to CTs with endosomal escape in VH or VL.^234^

Recent studies have identified a group of highly active CPPs that enter cells through endocytosis. Unlike most previous CPPs, they can effectively escape from the early endosomes into the cytoplasm.^235^ The specific mechanism is that cyclic CPPs combined with the endosomal membrane form a lipid structure domain including abundant CPPs. The domain from the endosome lipid structure is formed by budding with CPPs vesicles, and the budding from the endosome membrane can be released into the cytoplasm. For the first time, it has been demonstrated that CPPs and a non‐peptidic cell‐penetrating molecule can boost vesicle budding and rupture mechanisms in mammal cells to escape from the endosome. It provides an idea for cancer treatment to improve the endosomal escape efficiency of CPPs. In addition, strategies to enhance the endosomal release of CPP cargoes have also been proposed.^235^ By screening previously reported peptides without normal cytotoxicity and capable of destroying microbial membranes, the endosomal escape efficiency of the ultra‐positively charged Aurein 1.2 peptides was found to be 5‐fold higher.^236^ The CPP L7EB1 enhances cellular uptake by inducing apoptosis in vitro in cell lines PanC‐1 and NCI‐H226 using A3B lactose microsomes (a biocompatible and biodegradable polymer nano‐micelle that accumulates in tumours in vivo by enhancing permeability and preserving EPR effects). The photosensitiser 5,10,15,20‐tetraflorophenyl (pentaflorophenyl) porphyrins can photo‐induce endosomal escape and effectively deliver ABCG2 siRNA into the cytoplasm to achieve gene silencing.^237^ Some well‐defined mechanisms are as follows.

#### 4.3.1 Poly‐CPPs

Polycationic peptide dendrimers can serve as effective delivery carriers to solve the problem of endosomal interception.^238^ Based on this study, poly CPP has become a hot topic in tumour endosomal escape. Fl‐TAT3 was found to deliver molecules to the cytoplasm in a direct binding or simple co‐incubation mode.^239^ The drug amiloride can inhibit this effect, suggesting that macrophagocytosis is necessary and that MCPP can release molecules from the lumen of the endocytic organelle. Overall, trimer MCPP seems to escape from endosomes more efficiently than monomeric and dimeric CPPs. However, MCPP shares structural similarities with multiple antigenic peptides (MAPS).^240^ Therefore, the use of MCPP may produce abnormal cellular immune responses.

An earlier study designed a model of polymeric TAT‐3TAT entering the cytoplasm of living human cells through the membrane of late endosomes. It demonstrated that 3TAT cells have higher osmotic activity than their monomeric TATs, reflecting the advantage of CPPs in endosomal escape.^241^

#### 4.3.2 Endosome escape mediated by PH‐dependent membrane‐active peptide CPPs

Conceptually, the PH‐dependent membrane‐active peptide (PMAP) CPP chimera has two independent functions: CPP functions in inducing effective endocytosis of cargo and PMAP functions in increasing endosomal escape. Several studies have emphasised how these combinations affect their intracellular transmission. Although cargo affects the PMAP activity, PMAP affects the transportation characteristics of CPP‐cargo complexes. In addition, destruction of the endosomal membrane requires the lipophilicity of PMAP, which may alter the transport and function of the accompanying cargo.^242^ Therefore, the ways to conjugate CPPs to achieve targeting delivery and minimising toxicity remain to be considered. Endosomal retention of cargoes fused with the PMAP‐CPP construction remains a problem even after endosomal cleavage. The preferred design is suggested to include a cracking joint between the PMAP‐CPP and its cargo.^243^ In principle, this design can increase endosomal escape and cytoplasmic transfer, providing a new direction for anti‐cancer therapy.

#### 4.3.3 Photochemical internalisation

Photochemical internalisation (PCI) is a method using light and photosensitisers to capture peptides or proteins for endosomal escape and release into the cytoplasm. Mediated PCI can be used in vivo to deliver drugs to cancer cells by irradiating light only to the targeting tissues, and PCI facilitates temporal and spatial control.^244^ CPP‐cargo photosensitiser complexes can enter the cytoplasm via the PCI. For instance, TAT‐U1A‐PS (Tat [CPP], U1A RNA‐binding protein [cargo] and PS) can deliver RNA into the cytoplasm under light‐dependent action, and TAT‐BIM‐PS (Tat [CPP], Bim [cargo] covalent complex and PS) can induce apoptosis in mammalian cells.^229^ These results can guide the design of CPP‐cargo‐PS conjugates and enable PCI and photodynamic therapy with a wide range of target functions.

#### 4.3.4 Lipid fusion

Escape requires contact and fusion of membrane lipids. CPP (3TAT) can cause aggregation and fusion of PG (phosphatidylglycerol) and bis (monoacylglycerol) phosphate (BMP) LUV (large unilamellar vesicles of late endosomes). The research explains how the CPP leads to the specific escape of late endosomes and the subsequent release of endosomally entrapped macromolecular cargoes. The CPP interacts with BMP, an anionic lipid found in late endosomes. The presence and abundance of BMPs are the main determinants of endosomal escape. BMP has the unique ability to encapsulate the CPP, which is an event that opens pores at contact sites.^241^ The 3TAT used in this experiment was applied in previous studies to design dfTAT to mediate endosomal leakage. The dfTAT is used to deliver proteins, peptides or small molecules, which is effective in the cytosol of the cell. More interestingly, the mechanism described in this study is different from other membrane translocation models, indicating that membrane translocation requires encapsulation of CPP between multiple bilayers in close contact, contrary to the idea that CPP spans a single bilayer. This view may explain the unresolved phenomenon of endosomal escape and become a tool for future cell delivery, providing the molecular basis.^245^

#### 4.3.5 Membrane‐disruptive peptides

In terms of antifungal treatment, MDP can increase the permeability of cell membranes through its unique membrane destruction mechanism. It improves the transmembrane efficiency of drugs and increases the sensitivity of cells to anti‐cancer agents. This function may represent a promising future direction in the design of anti‐cancer treatment. In addition, the combination of MDPs and chemotherapeutic drugs can treat cancer.^246^ Melittin is a naturally occurring membrane cleavage peptide that can act as a transduction domain for peptides in the development of peptide‐mediated transfection.^247^ Moreover, the combination of melittin and docetaxel can reside in breast cancer cells.^246^ In a word, membrane cleavage peptide may be a suitable assistant for tumour therapy drug delivery similar to CPP, facilitating endosomal escape and even possessing anti‐cancer effects.

### 4.4 Optimisation of the immunogenicity and cytotoxicity

Due to their polypeptide properties, CPPs can increase the risk of adverse immune reactions in patients, resulting in lower drug effects and adverse immune response.^248^ Although the immunogenicity of CPPs is defective in anti‐cancer therapy, some studies have exploited the immunogenicity of CPPs and demonstrated their contribution to tumour immunotherapy. CPPs linked to antigens encapsulated in nano‐vaccines can influence intracellular localisation of antigens in time and space, promote cross‐presentation of antigens and stimulate antigen‐specific immune responses, especially CTL responses. Therefore, CPP modification to antigens is an effective strategy to enhance the efficiency of nano‐vaccines in cancer immunotherapy.^249^ CPPs‐PepFects (PFs) have demonstrated efficient NA delivery in both in vitro and in vivo experiments, making it important to assess their possible toxic effects. The cytotoxicity and immunogenicity of PF3, PF4 and PF6 peptides have been analysed in mononuclear leukaemia and peripheral blood mononuclear cell lines. In addition, the most widely used transfection reagents were analysed, including TP10, TAT, stearyl‐(RxR)4 polypeptides, Lipofectamine 2000 and Lipofectamine RNAiMAX.^250^ Suhorutsenko^251^ linked the potential toxicity and immunogenicity to PF peptides and compared them to the widely used cationic CPP, stearyl‐(RxR)4 polypeptides and the amphiphilic TP10. This study proposed that the charge and mol ratio in the peptide/NA complex is a possible factor. The complex has been shown to be stable in serum and highly effective in transfecting different cell lines.^250^ The results showed that after 24 or 48 h of incubation with PF polypeptide, the viability of THP‐1 cell lines was reduced without showing primary inflammatory effects. Even at low concentrations of 10 μM, PF peptide had no toxic effect on THP‐1 cell viability. In addition, liposome agents showed strong cytotoxicity, decreasing cell viability by up to 55%. IL‐1 β, IL‐18 and TNF‐α were not overexpressed in THP‐1 cells treated with TP10, TAT, stearyl‐(RxR)4 polypeptide or PF peptide. Similarly, no evidence of enhanced IL‐1 β production in vitro was observed in non‐cancer PBMC cell lines. According to the different expression of IL‐1 β, researchers estimated that PF peptide may be a factor triggering apoptosis of immune cells. Caspase‐1 and caspase‐3 were also evaluated in this study. PF3, PF4, PF6 polypeptides and PF peptides obtained by chemical modification of TP10 peptide sequence showed similar effects to TP10, TAT and stearoyl‐(RxR)4 polypeptides, with no effect on the host system. These peptides have no evidence of inflammatory effects in vivo and may be one of the keys to avoiding immunogenicity in anti‐cancer therapy.^252^

Several independent experiments on cell viability, membrane integrity and toxicity showed that amphiphilic peptides such as Transportan 10 could affect cell metabolism in 5–10 μM. In contrast, cationic CPPs, osmotin, TAT and non‐arginine did not have toxic effects in the same range. As PF peptides are designed based on TP10 peptide sequences, the possibility of inducing adverse toxic and inflammatory effects in vivo and in vitro cannot be excluded. Overall, the use of TAT does not induce a significant immune response, but long‐term toxicity remains unclear.^252^

Recent studies have also shown that a novel CPP, LDP12, can be used to internalise the HIV‐1 Nef‐MPER‐V3 protein into eukaryotic cell lines without toxicity, and its immunogenicity evaluation is currently being investigated. It is a promising CPP with the potential to further lessen immunogenicity problems.^253^

## 5 STRATEGIES FOR ANTI‐TUMOUR IMMUNOTHERAPY

So far, antibodies have played a vital role in treating various diseases, especially cancer. However, because they cannot penetrate the cell membrane to target intracellular molecules, some enter cells but hardly achieve the desired effect because they are also cleared by cellular efflux and lysosomal dissolution. For tumour immunotherapy, new advances have been made in cell‐penetrating antibodies, but further tests and determination of the optimal dose are needed. Thus, the most effective antagonistic or excitatory antibodies can be screened for beneficial targets to develop antibodies fitting the human sequence.^254^ Aftabizadeh et al.^255^ developed a therapeutic peptide that can effectively and specifically target cancer. They demonstrated that systemic injection of cell‐penetrating C‐MYC and Gp130 peptides could prevent pancreatic cancer growth and induce the anti‐tumour immune response and indirectly producing anti‐tumour effects through anti‐tumour immunity response. In addition, linking thio‐phosphorylated DNA oligonucleotides to antibodies allows their cells to penetrate tumour cells. Efficient cellular internalisation is achieved without interfering with antigen recognition by antibodies, leading to target blocking and regulation of target downstream genes. This modification enables antibodies to detect and regulate intracellular molecules in vivo and in vitro, providing the basis for immunotherapy of tumours.^254^ Although the critical function of MYC in human cancer makes it an ideal target for therapeutic interventions, long‐term inhibition of MYC has been considered infeasible. These experiments have proved the importance of CPP combination and conjugation in tumour immunotherapy. More effective CPPs also need to be developed in the future to better control difficult‐to‐transfect cells (e.g. stem cells, monocytes and APCs). In the context of the COVID‐19, using CPPs as a vaccine vector is costly and requires the transduction or transfection of immune cells.

**FIGURE 6:**
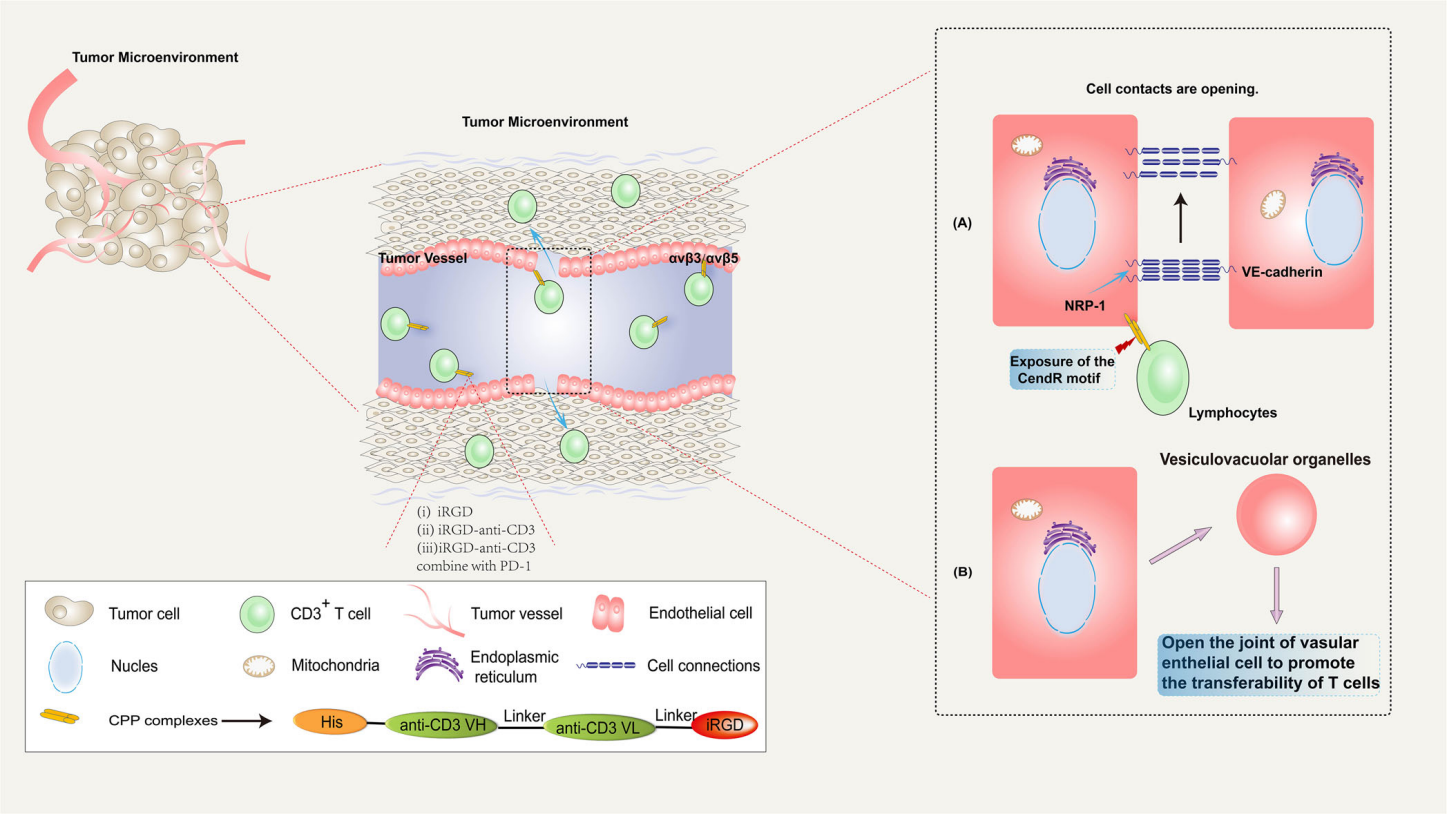
Mechanisms of CPP act in immunotherapy. Circulating iRGD‐modified T cells are tethered and rolled in blood flow by the engagement of αvβ3/αvβ5 expressed on tumour vascular endothelial cells. There are three CPP complexes: (i) IRGD (Lowest curative effect); (ii) iRGD‐anti‐CD3 (Medium curative effect) and (iii) iRGD‐anti‐CD3 combine with PD‐1. (Best curative effect) The key to facilitating tumour infiltration is to promote the opening of cell connections. There are two main mechanisms: (A) The interaction also initiates the proteolysis of iRGD and expose the CendR motif. The truncated peptide then binds to NRP‐1, triggering the tyrosine phosphorylation of VE‐cadherin and the formation of intercellular gaps. (B) Another vesicular transport pathway in the endothelial cytoplasm is termed vesiculovacuolar organelles. Then, the connected lymphocytes cross the vessel wall and infiltrate into the tumour parenchyma

Recently, a new research experiment has proposed an innovative point about immunotherapy: drugs can target both PD‐1 and PD‐L1 sites. The primary technologies used are organismal and single‐cell sequencing, which are also worth attending. Part of the mechanism is that down‐regulation of BRD1 reduces some immune cell dysfunctions driven by the tumour microenvironment, thereby improving the efficacy of immunotherapy.^256^ In addition, BsAbs is a novel immunotherapy with a primary strategy to recruit and activate T cells by simultaneously targeting the CD3 domain of the TCR complex and antigens abnormally expressed on the surface of tumour cells.^257^ The main progress of BsAbs before 2018 can be seen in this review.^258^ The use of the CPP‐iRGD is the key to facilitating tumour penetration and T‐cell activation. In 2021, bifunctional iRGD‐anti‐CD3 was found to enhance the anti‐tumour potency of T cells by facilitating tumour infiltration and T‐cell activation.^259^ Furthermore, the combination therapy with iRGD‐antiCD3 and PD‐1 blockade enhances the anti‐tumour capacity of cord blood‐derived T Cells.^260^ The mechanism of iRGD is illustrated in Figure 6. The development of more effective CPPs will be the focus of future research, providing a new direction for the therapy of tumours.

## 6 CONCLUSIONS AND PROSPECTS

Over the past 30 years, tremendous efforts have been made to discover novel cancer therapies, and many new anti‐cancer drugs have come to our attention. The discovery of CPPs greatly facilitates the development of DDDs, which have gained increasing attention for their ability to deliver many macromolecular therapeutic drugs with different properties into cells, especially in tumour therapy. The poor stability can be solved by chemical modification, conformational change and barrier strategy. ACPPs, tumour‐homing CPPs, antibody combined delivery, receptor or ligand combined delivery and passive targeting with intracellular specificity are proven to improve the specificity of CPPs. In terms of efficiency improvement, poly‐CPPs, endosomal escape mediated by PMAP CPPs, PCI, lipid fusion, proton sponge and disruptive membrane peptides can be the options. Certain anti‐cancer drugs based on CPPs are currently in the clinical experimental stage (as shown in Table 4). However, no such treatment method has been approved by FDA because of its drawbacks. In addition, the lack of a more comprehensive understanding of CPPs, such as their cellular uptake mechanisms, also imposes restrictions on their use in anti‐cancer therapy. The lack of a better understanding of structural and environmental factors affecting their activity under physiological conditions also hinders the progress of clinical trials. Current clinical trials are in the state of studying drug efficiency, whereas in contrast to small‐size drugs, classical adsorption, distribution, metabolism and excretion (ADME) pharmacokinetic issues play a very important role in peptide drugs. Timing, dosing and modes of administration are the main focus of later clinical trials. The transport efficiency, the cytotoxicity of each CPP cargo complex and required auxiliaries must be estimated for each target. These properties differ among CPPs, cargoes and cell types. All of these are underpinned by time and adequate clinical trials.

**TABLE 4:** Some CPPs‐based anti‐cancer therapies under clinical development

CPPs	Cargoes	Drugs in the trial	Therapeutic application	Status	ClinicalTrial.gov ID
A highly charged oligopeptide of human origin	SN38	SN38 alone	Tumour	Phase 1	NA
P28	P28	P28 alone	Solid tumours that resist standard methods of treatment	Phase I completed in 2014	NCT00914914
ACPPs	Cy5 and Cy7	AVB‐620	Tumour imaging	Phase 1 completed in 2017	NCT02391194
P28	Non‐HDM2‐ mediated peptide inhibitor of P53	azurin‐derived CPP p28	Central nervous system tumours	Phase I completed in 2013	NCT01975116
ALRN‐6924	Palbociclib	ALRN‐6924 alone and in combination With palbociclib	Solid tumour, lymphoma and peripheral T‐cell lymphoma	Phase 2a completed in 2020	NCT02264613
ALRN‐6924	Cytarabine	ALRN‐6924 alone and in combination with cytarabine	Acute myeloid leukemia and advanced myelodysplastic syndrome	Phase 1 completed in 2019	NCT02909972
ALRN‐6924	Paclitaxel	ALRN‐692 in combination with paclitaxel	Advanced, metastatic or unresectable solid tumours	Phase 1	NCT03725436
ALRN‐6924	Cytarabine	ALRN‐6924 alone or in combination with cytarabine	leukemia, pediatric brain tumour, pediatric solid tumour, pediatric lymphoma	Phase 1	NCT03654716
ALRN‐6924	Topotecan	Phase 1b ALRN‐6924 with topotecan Phase 2 topotecan alone and in combination with ALRN‐6924	Small cell lung cancer	Phase 1a completed in 2019 Phase 1b Phase 2	NCT04022876
BT1718	–	BT1718 alone	Advanced solid tumours, non‐small cell lung cancer, non‐small cell lung sarcoma and esophageal cancer	Phase 1 Phase 2	NCT03486730
PEP‐010	Paclitaxel	PEP‐010 alone PEP‐010 in combination with paclitaxel	Metastatic solid tumour cancer	Phase 1	NCT04733027
ATP128	BI 754091	ATP128 alone and in combination with BI 754091	Stage IV colorectal cancer	Phase 1a completed in 2020 Phase 1b	NCT04046445

This review included many pre‐clinical and clinical research progresses, showing some CPPs’ major advantages as determinant molecules for cellular internalisation and tumour targeting. In addition, some defects that restrict the further application of CPPs in tumour treatment are summarised, such as lack of cell specificity, short action time and poor stability in vivo. The above strategies have successfully improved the defects of CPPs during in vitro experiments, and some of them have achieved satisfactory results in pre‐clinical animal models. By summarising the role of CPP on potential anti‐cancer strategy, CPPs can be more eligible for novel anti‐cancer pharmaceutics soon. The optimisation strategy gaps and other drawbacks remain challenges to be overcome in the future.

Notably, computational methods are receiving increasing attention in predicting novel CPP sequences.^261^, ^262^, ^263^ Substantial efforts have been made to design and modulate novel CPP sequences, exhibiting increased membrane permeability and improved target specificity. Quantitative analysis tracking non‐fragmented peptide drugs has a high possibility to contribute to the development of CPP‐based therapeutic. Development in peptidomimetics, stealth techniques, high‐throughput selective ligand screening, bio‐orthogonal conjugation and biodegradable bonds may also facilitate the creation of the next generation of CPPs. Focusing on uptake mechanisms and exploring the relationship between properties of distinct CPPs, cell types and cargoes can enable the theoretical prediction of optimal partners and conditions for a given task. With the special probabilities of CPPs for internalising antibodies, enzymes, chaperons and other large functional proteins into live cells, new therapeutics can be developed, which may support the further development of clinical trials based on CPPs.

## COMPETING INTERESTS

The authors declare that they have no competing interests.
